# Lithium Aspartate for Long COVID Fatigue and Cognitive Dysfunction: A Randomized Clinical Trial

**DOI:** 10.1001/jamanetworkopen.2024.36874

**Published:** 2024-10-02

**Authors:** Thomas Guttuso, Jingtao Zhu, Gregory E. Wilding

**Affiliations:** 1 Department of Neurology, Jacobs School of Medicine and Biomedical Sciences, University at Buffalo, Williamsville, New York; 2 Department of Biostatistics, Jacobs School of Medicine and Biomedical Sciences, University at Buffalo, Williamsville, New York

## Abstract

**Importance:**

Neurologic post–COVID-19 condition (PCC), or long COVID, symptoms of fatigue and cognitive dysfunction continue to affect millions of people who have been infected with SARS-CoV-2. There currently are no effective evidence-based therapies available for treating neurologic PCC.

**Objective:**

To assess the effects of lithium aspartate therapy on PCC fatigue and cognitive dysfunction.

**Design, Setting, and Participants:**

A randomized, double-blind, placebo-controlled trial (RCT) enrolling participants in a neurology clinic from November 28, 2022, to June 29, 2023, with 3 weeks of follow-up, was conducted. Subsequently, an open-label lithium dose-finding study with 6 weeks of follow-up was performed among the same participants enrolled in the RCT. Eligible individuals needed to report new, bothersome fatigue or cognitive dysfunction persisting for more than 4 weeks after a self-reported positive test for COVID-19, Fatigue Severity Scale-7 (FSS-7) or Brain Fog Severity Scale (BFSS) score of 28 or greater, Beck Depression Inventory-II score less than 29, and no history of a condition known to cause fatigue or cognitive dysfunction. All participants in the RCT were eligible for the dose-finding study, except for those who responded to the placebo. Intention-to-treat analysis was used.

**Intervention:**

Lithium aspartate, 10 to 15 mg/d, or identically appearing placebo for 3 weeks followed by open-label lithium aspartate, 10 to 15 mg/d, for 2 weeks. In the subsequent dose-finding study, open-label lithium aspartate dosages up to 45 mg/d for 6 weeks were given.

**Main Outcomes and Measures:**

Change in sum of FSS-7 and BFSS scores. The scores for each measure range from 7 to 49, with higher scores indicating more severe symptoms. Secondary outcomes included changes from baseline in the scores of additional questionnaires.

**Results:**

Fifty-two participants were enrolled (30 [58%] males; mean [SD] age, 58.54 [14.34] years) and 26 were randomized to treatment with lithium aspartate (10 females) and 26 to placebo (12 female). Two participants assigned to lithium aspartate were lost to follow-up and none withdrew. No adverse events were attributable to lithium therapy. There were no significant intergroup differences for the primary outcome (−3.6; 95% CI, −16.6 to 9.5; *P* = .59) or any secondary outcomes. Among 3 patients completing a subsequent dose-finding study, open-label lithium aspartate, 40 to 45 mg/d, was associated with numerically greater reductions in fatigue and cognitive dysfunction scores than 15 mg/d, particularly in 2 patients with serum lithium levels of 0.18 and 0.49 mEq/L compared with 1 patient with a level of 0.10 mEq/L.

**Conclusions and Relevance:**

In this RCT, therapy with lithium aspartate, 10 to 15 mg/d, was ineffective for neurologic PCC fatigue and cognitive dysfunction. Another RCT is required to assess the potential benefits of higher lithium dosages for treating neurologic PCC.

**Trial Registration:**

ClinicalTrials.gov Identifier: NCT05618587 and NCT06108297

## Key Points

**Question:**

Is lithium aspartate effective for treating neurologic post–COVID-19 condition fatigue and cognitive dysfunction?

**Findings:**

In a randomized clinical trial including 52 participants, lithium aspartate, 10 to 15 mg/d, for 3 weeks provided no significant improvements to fatigue or cognitive dysfunction scores. A subsequent dose-finding study found open-label lithium aspartate, 40 to 45 mg/d, to be associated with numerically greater symptomatic benefit, particularly in 2 patients with serum lithium levels of 0.18 and 0.49 mEq/L, compared with 1 patient with a level of 0.10 mEq/L.

**Meaning:**

The findings of this trial suggest that lithium aspartate, 10 to 15 mg/d, is ineffective for neurologic post–COVID-19 condition fatigue and cognitive dysfunction; the effect of higher dosages needs to be assessed in another randomized clinical trial.

## Teaser

This randomized clinical trial and subsequent dose-finding study examine the use and dosage of lithium aspartate therapy in patients with neurologic post-COVID condition symptoms of fatigue and cognitive dysfunction.

## Introduction

Post–COVID-19 condition (PCC; also known as long COVID) is the persistence of symptoms for at least 4 weeks after recovery from a COVID-19 infection.^1^ PCC symptoms last for more than 6 months in approximately 10% of patients, with fatigue, postexertional malaise, and cognitive dysfunction being the most common.^2,3^ Due to the lack of effective treatments, PCC continues to cause major disability and reduced quality of life in an estimated 65 million people worldwide.^4^ PCC can also cause substantial financial consequences, with about 45% of patients requiring reduced working hours and about 22% of patients unable to be employed.^3^

There are both autopsy and neuroimaging data supporting chronic brain inflammation as a mechanistic contributor to the neurologic PCC symptoms of fatigue and cognitive dysfunction.^5,6,7,8^ Because patients coming to autopsy typically had severe systemic illness that may influence brain pathologic changes in ways distinct from those in patients living with neurologic PCC, data from neuroimaging studies may better identify the mechanisms involved and guide the development of future effective therapies.

The brain glial cells of microglia and astrocytes are primary mediators of neuroinflammation when activated.^9^ Using 2 different positron emission tomography (PET) ligands, widespread increases in microglial and astrocyte activation are seen in patients with neurologic PCC compared with healthy controls, signifying increased neuroinflammation.^7,8^ These studies show the greatest increases in inflammation in the ventral striatum, dorsal putamen, and thalamus, which are regions previously implicated in engendering symptoms of fatigue and cognitive dysfunction.^10,11,12,13^ Because inflammation seen on PET imaging is known to localize to brain sites preferentially affected in neurologic diseases, including Alzheimer disease, Parkinson disease, and stroke,^14,15,16,17^ therapies known to suppress glial activation and neuroinflammation in these sites would represent promising therapies for neurologic PCC fatigue and cognitive dysfunction.

Lithium, in addition to being an effective treatment for bipolar disorder, has a multitude of neuroprotective actions.^18^ One of these actions is its ability to reduce neuroinflammation by suppressing both microglial and astrocytic activation.^19,20,21,22,23^ In a pilot clinical trial in patients with Parkinson disease, lithium aspartate, 45 mg/d, was associated with reductions in a magnetic resonance imaging (MRI) neuroinflammation biomarker called free water in several brain sites, including the thalamus.^24^ Due to these actions, lithium was theorized to be a potential treatment for PCC fatigue and cognitive dysfunction. After 9 of 10 patients with neurologic PCC under the care of one of us (T.G.) reported satisfactory benefits to fatigue and/or cognitive dysfunction within 3 to 5 days after starting lithium aspartate, 5 to 10 mg/d, and 5 patients subsequently reported additional benefits at 15 mg/d, a randomized clinical trial (RCT) was performed.

## Methods

A randomized, double-blind, placebo-controlled, parallel-group trial was performed at the University at Buffalo with patient enrollment from November 28, 2022, to June 29, 2023. The university’s institutional review board approved the study before patient enrollment. All patients provided written informed consent; no financial compensation was provided. The trial protocol is included in Supplement 1. This study followed the Consolidated Standards of Reporting Trials (CONSORT) reporting guideline.

Patients were recruited through the University at Buffalo Western New York Community-Based Long COVID Registry, local newspaper advertisements, and primary care offices. Patients were eligible for enrollment if they reported having a positive test for COVID-19 with subsequent new and bothersome fatigue and/or cognitive dysfunction symptoms for more than 4 weeks after recovering from the acute infection; no tobacco or tetrahydrocannabinol intake for more than 6 months^25^; no current or history of lithium use; no change in psychoactive or steroid medications for 30 days or more; no history of fibromyalgia, chronic fatigue syndrome, rheumatoid arthritis, or other conditions known to be associated with fatigue or cognitive dysfunction before testing positive for COVID-19; not applying for or receiving disability payments for PCC; and not pregnant or nursing. At the screening visit, patients needed to have a Fatigue Severity Scale 7-item version (FSS-7)^26,27^ score or Brain Fog Severity Scale (BFSS) score of 28 or higher, a Beck Depression Inventory-II (BDI-II) score lower than 29, and a negative urine pregnancy test if female of child-bearing potential. Due to the lack of a validated cognitive dysfunction scale, a BFSS was developed for this study, using the same 7 questions as the FSS-7 but with brain fog (cognitive dysfunction) substituted for fatigue for each question. The instructions for the BFSS stated “brain fog is difficulty with concentration, word-finding, organization, and/or short-term memory sometimes causing a person to feel like they are in a haze and/or confused when trying to complete simple tasks.” Change in sum of the FSS-7 and BFSS scores was the primary outcome of the study. Secondary outcomes were changes from baseline in additional questionnaires. These included the Headache and Body Pain Bother Scale (each 5-point frequency Likert scales), Generalized Anxiety Disorder Scale-2 (GAD-2),^28^ Short Form-12 Health Survey (SF-12)^29^ modified to reflect previous 1 week of symptoms, Well Being Scale (WBS)^30^ and the Insomnia Severity Index (ISI).^31^ Also, the Digit Symbol Substitution^32^ and Delayed Recall Tests from the Montreal Cognitive Assessment,^33^ versions 1 and 2, were administered. The FSS-7 and BFSS have score ranges of 7 to 49; Modified Fatigue Impact Scale (MFIS) range is 0 to 84; Perceived Deficits Questionnaire 5-Item Version (PDQ-5), 0 to 20; BDI-II, 0 to 63; and GAD-2, 0 to 6. Higher scores for each measure indicate more severe symptoms. Scores for the other measures included SF-12 (scores >50 indicate better-than-average health-related quality of life), Patient Global Impression of Change (PGIC) (score range, 1-7, with higher scores indicating greater improvement and lower scores indicating greater worsening of symptoms, and WBS-Well Being Scale (score range, 0-10, with higher scores indicating greater sense of well-being). Race and ethnicity were self-reported by study participants and were assessed to help with interpretation of the generalizability of the results across races and ethnicity. 

Eligible patients were randomly assigned (1:1) to receive overencapsulated lithium aspartate capsules, each containing 5 mg of elemental lithium or identically appearing, overencapsulated placebo capsules (filled with microcrystalline cellulose). Lithium aspartate was chosen over lithium carbonate and lithium orotate because lithium aspartate was the lithium salt used by the 10 patients with PCC previously treated by one of us, is readily available as a dietary supplement to maximize patient accessibility, and because orotate increases the occurrence of several cancers in animal models.^34,35,36^ The randomization table was devised by the study’s biostatistician (G.E.W.). Study pill bottles were labeled with sequential randomization ID numbers by the research pharmacy and patients were assigned pill bottles sequentially in the order of enrollment. All clinical study team members remained blinded to treatment allocations until all patients completed the study and all primary and secondary outcome measure analyses were completed by the biostatistician.

Patients were instructed to take 2 capsules per day for the initial 10 days. If their PCC symptoms were still bothersome, they could increase the dosage to 3 capsules per day for the last 11 days. A follow-up visit was made 21 days after the baseline visit. Baseline questionnaires/tests were again assessed in addition to the PGIC,^37^ Desire to Continue Therapy, and Change in Sense of Smell and Taste Scale (7-point Likert scale with higher scores indicating greater improvement and lower scores indicating worsening of symptoms) questionnaires. Patients were then provided with a 2-week supply of open-label lithium aspartate, 5-mg, capsules with instructions to start with 2 capsules per day for 7 days and then 3 capsules per day for 7 days if still bothered by PCC symptoms. Patients were provided with a copy of all but 2 of the questionnaires (Digit Symbol Substitution and Delayed Recall tests) and instructed to complete them after 2 weeks and mail them to the study team in a provided self-addressed stamped envelope.

### Dose-Finding Study

After completing the double-blind study, 1 patient who obtained additional lithium aspartate from a dietary supplement vendor informed the investigator of experiencing satisfactory benefit to fatigue and cognitive dysfunction after slowly increasing the dosage from 15 to 40 mg/d over a 5-week period, resulting in a serum lithium level of 0.17 mEq/L (to convert to millimoles per liter, multiply by 1). During the double-blind phase, while receiving placebo, and the open-label phase with lithium aspartate, 15 mg/d, this patient experienced no improvements in fatigue or cognitive dysfunction based on the FSS-7 and BFSS scores. Because neuroinflammation is implicated in PCC fatigue and cognitive dysfunction and preliminary results found lithium aspartate, 45 mg/d, to have more consistent and robust reductions in the brain inflammation biomarker free water compared with lithium aspartate, 15 mg/d, or lithium carbonate, approximately 150 mg/d,^24^ we theorized that lithium aspartate, 45 mg/d, may be more effective for neurologic PCC than 15 mg/d.

After completion of the RCT, a dose-finding study was initiated to assess whether open-label lithium aspartate dosages up to 45 mg/d were associated with greater reductions in FSS-7 or BFSS scores compared with 15 mg/d in individual patients from the RCT and to attempt to identify a therapeutic serum lithium level akin to how lithium is used to treat bipolar disorder—its only US Food and Drug Administration–approved indication.^38^ Such data could help determine whether further research was merited using lithium therapy for PCC and help guide the design of a future trial.

After obtaining institutional review board approval from the University at Buffalo, patients from the original RCT were invited to participate in the dose-finding study. The study protocol is included in Supplement 2. Patients were eligible if they were not a placebo responder based on the responder analysis results (<18-point reduction in FSS-7 or <15-point reduction in BFSS if receiving placebo during the double-blind study phase), had FSS-7 or BFSS scores of 28 or higher, or FSS-7 and BFSS scores lower than 28 and a PGIC score of 6 or 7 while still receiving lithium aspartate at the baseline visit. After providing written informed consent, patients completed the FSS-7, BFSS, Headache and Body Pain Bother Scale, GAD-2, SF-12, WBS, and ISI in addition to the MFIS^39^ and the PDQ-5,^40^ with both the MFIS and PDQ-5 modified to reflect symptoms over the previous week. A venous blood sample was assessed for thyroid-stimulating hormone, lithium level, and chemistry panel. Patients were instructed to take lithium aspartate, 5-mg, capsules, 2 capsules twice daily for a week. Every week thereafter, the daily dosage was increased by 1 capsule a day up to a maximum dosage of 4 capsules every morning and 5 capsules every night at bedtime (45 mg/d), as tolerated. A follow-up visit occurred 3 weeks after each patient achieved a maximum tolerated dosage when the same questionnaires and blood tests were assessed. Serum trough lithium levels were assessed 10 to 14 hours after the bedtime dose. Patients were then provided with a 3-week supply of lithium aspartate capsules and a copy of the questionnaires and instructed to complete them after 3 weeks and mail them to the study team in a provided self-addressed, stamped envelope.

### Statistical Analysis

Based on the anecdotal reports from the 10 patients with PCC treated with lithium aspartate, 5 to 15 mg/d, we estimated a 40% intergroup difference for the primary end point and a 48% SD for the placebo group. With these estimations, a total of 50 patients was needed to detect an intergroup difference with 80% power at a 2-tailed α level of .05.

Frequencies and relative frequencies were used to summarize binary variables, and numeric variables were summarized by the mean (SD). Numeric changes in study outcomes between groups were statistically assessed using standard *t* tests. Group differences in binary outcomes were tested using the Barnard unconditional exact test and intention-to-treat analysis. All tests were 2-sided and performed in conjunction with a *P* < .05 nominal significance level. All analyses were conducted using SAS, version 9.4 statistical software (SAS Institute LLC).

Responder analyses compared intergroup differences in the percentage of patients achieving FSS-7 or BFSS score reductions of at least the mean for all patients recording a PGIC score of 6 (much improved) or 7 (very much improved).

## Results

### Double-Blind Study

From November 28, 2022, to June 29, 2023, 251 patients were screened, of whom 52 (30 males [58%]; 22 females [42%]) were eligible, enrolled, and randomized (Figure). Mean (SD) age was 58.54 (14.34) years. Patients in the lithium and placebo groups were well matched (Table 1). Two patients (both receiving lithium) were lost-to-follow up and none withdrew from the study. All 50 patients who completed the double-blind phase entered the open-label lithium phase, and 45 provided outcome data (Figure).

**Figure.**
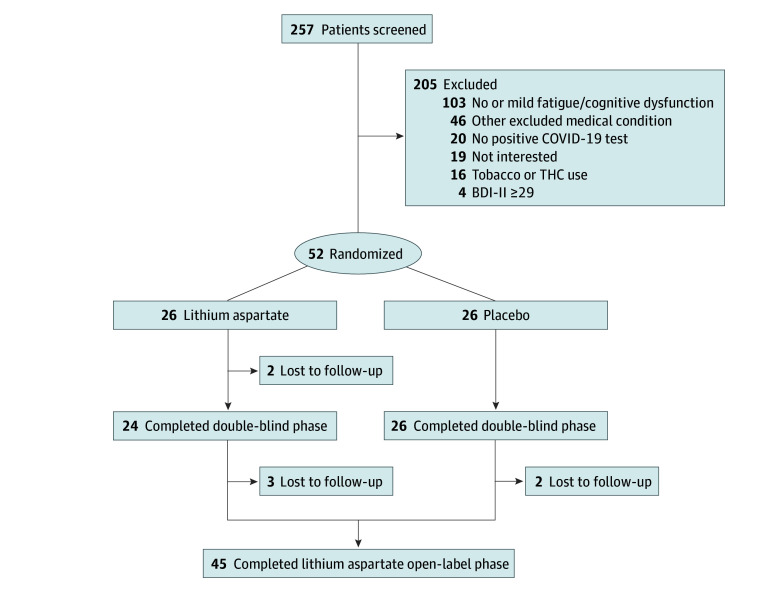
Patient Flowchart BDI-II indicates Beck Depression Inventory-II; THC, tetrahydrocannabinol.

**Table 1.** Baseline Characteristics

Participant and scale characteristics	Mean (SD)	
	Lithium aspartate (n = 24)	Placebo (n = 26)
**Participant**		
Age, y	62.3 (14.3)	55.0 (13.7)
Sex, No. (%)		
Male	14 (58)	14 (54)
Female	10 (42)	12 (46)
Race and ethnicity, No. (%)^a^		
Hispanic White	2 (8)	0
Non-Hispanic White	22 (92)	26 (100)
Received any COVID-19 vaccinations, No. (%)	20 (83)	25 (96)
Duration of PCC, mo	17.4 (9.9)	17.5 (10.4)
**Scale**		
FSS-7^b^	39.5 (10.9)	43.0 (8.0)
BFSS^c^	37.3 (9.7)	35.3 (10.9)
Sum of FSS-7 and BFSS	76.7 (17.2)	78.4 (13.7)
Insomnia Severity Scale^d^	13.0 (7.2)	14.4 (6.9)
Generalized Anxiety Scale-2^e^	1.7 (1.7)	2.3 (1.7)
Beck Depression Inventory-II^f^	14.8 (7.1)	17.2 (6.6)
Short Form-12 Health Survey: Physical Component score^g^	33.9 (9.7)	34.0 (11.2)
Short Form-12 Health Survey: Mental Component score^h^	43.4 (9.0)	43.4 10.9)
Well-Being Scale^i^	5.0 (1.9)	4.5 (2.0)
Digit Symbol Substitution Test^j^	47.2 (8.9)	50.2 (14.6)
5 Object Registration^k^	4.6 (0.6)	4.7 (0.6)
5 Object 3-Minute Recall^l^	4.0 (0.9)	4.4 (0.8)
Headache Bother Questionnaire^m^	2.7 (1.2)	2.7 (1.3)
Body Pain Bother Questionnaire^n^	3.5 (1.2)	3.1 (1.4)
Sense of Smell and Taste Impairment Scale^o^	2.3 (1.5)	1.6 (0.9)

Abbreviations: BFSS, Brain Fog Severity Scale; FSS-7, Fatigue Severity Scale-7; PCC, post–COVID-19 condition.

^a^ Race and ethnicity were assessed to help with interpretation of the generalizability of the results across races and ethnicity. All races were assessed but 100% of enrolled patients were White.

^b^ FSS-7 score range, 7 to 49; higher scores indicate more severe symptoms.

^c^ BFSS score range, 7 to 49; higher scores indicate more severe symptoms.

^d^ Insomnia Severity Index; range 0-28, with higher scores indicating more severe insomnia.

^e^ Generalized Anxiety Disorder Scale-2 score range, 0 to 6; higher scores indicate more severe symptoms.

^f^ Beck Depression Inventory-II score range, 0 to 63; higher scores indicate more severe symptoms.

^g^ Short Form-12 Health Survey: Physical Component scores greater than 50 indicate better-than-average health-related quality of life.

^h^ Short Form-12 Health Survey: Physical Component scores greater than 50 indicate better-than-average health-related quality of life.

^i^ Well-Being Scale scores range from 0 to 10; higher scores indicate greater sense of well-being.

^j^ Digit Symbol Substitution Test score range, 0-93; higher scores indicate better cognition.

^k^ 5 Object Registration score range, 0-5, higher scores indicate better cognition.

^l^ 5 Object 3-Minute Recall score range, 0-5, higher scores indicate better cognition.

^m^ Headache Bother Questionnaire score range, 1-5; higher scores indicate worse symptoms.

^n^ Body Pain Bother Questionnaire score range, 1-5; higher scores indicate worse symptoms.

^o^ Sense of Smell and Taste Impairment Scale score range, 1-5; higher scores indicate worse symptoms.

In the double-blind phase, 4 patients receiving placebo and 6 receiving lithium aspartate opted to remain at the 2 capsules per day dosage for the entire double-blind phase. Five patients receiving placebo and 2 receiving lithium aspartate reported treatment-emergent adverse events. One patient receiving lithium aspartate with a history of recurring leg cellulitis experienced leg cellulitis that resolved with oral antibiotics. Another patient receiving lithium experienced a foot fracture from an accident. No patients reported treatment-emergent adverse events during the open-label lithium study phase.

Results showed no significant intergroup differences for the primary outcome (−3.6; 95% CI, −16.6 to 9.5; *P* = .59) or any of the secondary outcome measures (Table 2). Responder analyses showed a mean (SD) FSS-7 score change of −17.4 (12.4) and BFSS score change of −14.4 (11.8) for all patients reporting to have much improved or very much improved symptoms on the PGIC. There were no significant intergroup differences for the percentage of responders achieving FSS or BFSS score reductions of at least these cutoff values.

**Table 2.** Results From Double-Blind Study

Outcome measure	Change from baseline,^a^ mean (SD)			*P* value
	Lithium aspartate (n = 24)	Placebo (n = 26)	Intergroup difference (95% CI)	
FSS-7	−11.3 (12.6)	−8.6 (12.3)	−2.7 (−9.7 to 4.4)	.46
BFSS	−9.0 (13.8)	−8.1 (10.6)	−0.9 (−7.9 to 6.0)	.79
Sum of FSS-7 and BFSS	−20.3 (24.7)	−16.7 (21.1)	−3.6 (−16.6 to 9.5)	.59
Insomnia Severity Index (with BL score ≥10)	−6.0 (6.7)	−4.4 (4.8)	−1.6 (−5.5 to 2.3)	.41
Generalized Anxiety Disorder Scale-2 (with BL score ≥1)	−0.8 (1.7)	−1.4 (1.7)	0.6 (−0.5 to 1.8)	.28
Beck Depression Inventory-II	−5.2 (7.5)	−5.6 (5.9)	0.4 (−3.5 to 4.2)	.85
Short-Form-12 Health Survey: PCS	5.1 (8.5)	4.2 (11.1)	0.9 (−4.8 to 6.6)	.75
Short-Form-12 Health Survey: MCS	8.0 (10.9)	5.8 (8.0)	2.2 (−3.3 to 7.6)	.43
Well-Being Scale	1.0 (2.0)	0.9 (2.1)	0.1 (−1.1 to 1.3)	.89
Digit Symbol Substitution Test	4.3 (5.9)	6.6 (9.0)	−2.3 (−6.7 to 2.1)	.29
5-Object Registration	0.1 (0.4)	0.0 (0.7)	0.1 (−0.2 to 0.4)	.61
5-Object 3-Minute Recall	0.5 (1.0)	0.3 (0.8)	0.2 (−0.4 to 0.6)	.67
Headache Bother Questionnaire (with BL score ≥2)	−0.6 (0.7)	−0.7 (1.2)	0.1 (−0.5 to 0.8)	.58
Body Pain Bother Questionnaire (with BL score ≥2)	−0.9 (1.2)	−1.0 (1.2)	0.1 (−5.3 to 0.7)	.77
Patient Global Impression of Change (at follow-up visit)	4.7 (1.1)	4.6 (1.2)	0.1 (−0.5 to 0.8)	.68
Desire to continue therapy (at follow-up visit), No. (%), yes	12 (50)	13 (50)	0.0 (−0.3 to 0.3)	1
Change in sense of smell and taste (at follow-up visit, with BL SOSTIS score ≥2)	4.1 (0.3)	4.2 (0.4)	−0.1 (−0.4 to 0.2)	.50

Abbreviations: BFSS, Brain Fog Severity Scale; BL, baseline; FSS-7, Fatigue Severity Scale-7; MCS, Mental Component Score; PCS, Physical Component Score; SOSTIS, Sense of Smell and Taste Impairment Scale.

^a^ The score ranges and explanations of the measures are given in the Table 1 footnotes except Patient Global Impression of Change (score range 1-7; higher scores indicate greater improvement and lower scores indicate greater worsening of symptoms).

### Dose-Finding Study

Of the 43 patients invited to participate in the dose-finding study who were not placebo responders during the double-blind phase, 5 enrolled from January 5 to 25, 2024. Patient baseline characteristics are summarized in Table 3. Patients 1, 2, 4, and 5 continued lithium aspartate, 15 mg/d, for 7 to 12 months after completing the double-blind study, but only patient 1 reported continued satisfactory benefit from this dosage on entering the dose-finding study. Patient 1 stated he had not tried to discontinue lithium therapy over the previous 12 months. Patient 3 had a history of intermittent diarrhea and withdrew from the study due to worsening diarrhea and no perceived improvements in fatigue or cognitive dysfunction at 40 mg/d. Patient 5 experienced mild sedation at 45 mg/d, which resolved at 40 mg/d. There were no clinically significant changes in serum thyroid-stimulating hormone levels or estimated glomerular filtration rates in any patient. Among 3 patients who completed the dose-finding study, open-label lithium aspartate, 40 to 45 mg/d, was associated with numerically greater reductions in fatigue and cognitive dysfunction scores than 15 mg/d, particularly in 2 patients with serum lithium levels of 0.18 and 0.49 mEq/L compared with 1 patient with a level of 0.10 mEq/L (Table 4). Seven months after enrolling in the dose-finding study, patient 1 reported to have discontinued lithium aspartate therapy without any recurrent subjective fatigue or cognitive dysfunction.

**Table 3.** Dose-Finding Study Patient Characteristics

Patient No., Sex	PCC duration, mo	Study baseline values^a^					
		Double-blind		Dose-finding			
		FSS-7	BFSS	FSS-7	BFSS	MFIS	PDQ-5
1, Male	7	36	14	15	7	20	4
2, Male	9	28	41	29	36	46	15
3, Male	24	41	35	39	33	50	11
4, Female	30	49	49	49	35	48	9
5, Female	29	49	37	49	18	43	10

Abbreviations: BFSS, Brain Fog Severity Scale; FSS-7, Fatigue Severity Scale-7; MFIS, Modified Fatigue Impact Scale; PCC, post–COVID-19 condition; PDQ-5, Perceived Deficits Questionnaire 5-Item Version.

^a^ The score ranges and explanations of the measures are given in the Table 1 footnotes.

**Table 4.** Dose-Finding Study Results^a^

Patient No.	Double-blind study, 15 mg/d, % change from baseline in placebo vs lithium		Lithium aspartate, 15 mg/d for 7-12 mo, % change from baseline		Lithium aspartate, 40-45 mg/d, for 6 wk, % change from start of dose-finding study				PGIC	WBS	Trough serum lithium level, mEq/L (dosage)
	FSS-7	BFSS	FSS-7	BFSS	FSS-7	BFSS	MFIS	PDQ-5			
1	6 Placebo	36 Placebo	−58	−50	NA	NA	NA	NA	7	7	<0.10 (15 mg/d)
2	−7 Placebo	−10 Placebo	4	−12	−17	−31	−20	−27	5	7	0.10 (45 mg/d)
4	−41 Lithium	−57 Lithium	0	−29	−29	−80	−65	−89	7	7	0.49 (45 mg/d)
5	−84 Lithium	−76 Lithium	0	−51	−86	−50	−70	−50	6	8	0.18 (40 mg/d)

Abbreviations: BFSS, Brain Fog Severity Scale; FSS-7, Fatigue Severity Scale-7; MFIS, Modified Fatigue Impact Scale; NA, not applicable; PDQ-5, Perceived Deficits Questionnaire, 5-item version; PGIC, Patient Global Impression of Change; WBS, Well Being Scale.

SI conversion factor: To convert lithium to millimoles per liter, multiply by 1.

^a^ The score ranges and explanations of the measures are given in the Table 1 footnotes. FSS-7 and BFSS maximum score decrease of 86%; PDQ-5 is reported as percent change; PGIC and WBS are single scores reported at the end of the dose-finding study.

## Discussion

The randomized, double-blind, placebo-controlled trial showed therapy with lithium aspartate, 10 to 15 mg/d, to be ineffective for treating PCC fatigue and cognitive dysfunction. These results highlight the importance of performing RCTs to assess the efficacy of potential PCC therapies before recommending their use. This RCT was pursued based on highly encouraging, albeit purely anecdotal, findings of satisfactory benefit to reduce PCC fatigue and/or cognitive dysfunction in 9 of 10 patients after starting lithium aspartate, 5 to 15 mg/d, in addition to the known brain anti-inflammatory actions of lithium.^19,20,21,22,23^ To our knowledge, the only pharmacotherapy that has shown benefit for neurologic PCC fatigue in an RCT is AXA1125, a therapy with anti-inflammatory actions,^41^ which is still in development and not currently available to patients.^42^ The antidepressant vortioxetine recently showed benefit for PCC cognitive dysfunction as a secondary outcome, but only in patients with increased serum C-reactive protein levels.^43^

Although our decision to pursue the subsequent lithium dose-finding study was also based on a single anecdotal report of satisfactory symptom reduction with lithium aspartate, 40 mg/d, we felt it was worthwhile because this patient had previously shown no benefit while receiving double-blind placebo or lithium aspartate, 15 mg/d, and higher lithium dosages had previously been associated with reductions in brain free water,^24^ a known neuroinflammation biomarker.^44,45,46^ However, only 5 patients from the double-blind trial participated in the dose-finding study, 4 of whom qualified for higher lithium dosage therapy. With such a small, open-label study subject to selection bias, it is difficult to draw any reliable conclusions regarding the merits of future neurologic PCC lithium trials. Nevertheless, results from 3 patients who completed the dose-finding study (patients 2, 4, and 5 in Table 4) and the anecdotal patient suggest that serum lithium levels between 0.18 and 0.49 mEq/L may offer meaningful improvements in PCC fatigue and/or cognitive dysfunction. Patients 4 and 5, who achieved these serum levels, reported large symptomatic improvements, particularly on the MFIS, PDQ-5, and PGIC scales, while patient 2 did not at a serum level of 0.10 mEq/L (Table 4). Changes in BFSS and PDQ-5 scores were very similar in these 3 patients, providing preliminary evidence of BFSS validity. Although patient 1 had sustained and satisfactory improvements in fatigue and cognitive dysfunction at a serum lithium level less than 0.10 mEq/L, because this patient eventually reported no symptom recurrence after discontinuing lithium therapy, the symptoms may have resolved irrespective of lithium therapy at the time of enrollment in the dose-finding study.

The possibility that lithium serum levels of approximately 0.18 to 0.49 mEq/L may be required for symptomatic benefit in neurologic PCC has support from clinical trial data in Alzheimer and Parkinson disease, disorders like neurologic PCC in which neuroinflammation is mechanistically implicated.^14,15^ In an RCT in patients with amnestic mild cognitive impairment, a precursor condition to Alzheimer disease, serum lithium levels of 0.25 to 0.50 mEq/L were associated with slowed cognitive decline and significantly reduced cerebrospinal fluid levels of the Alzheimer disease biomarker p-tau.^47^ In a previous lithium Parkinson disease biomarker pilot trial, 4 patients with serum lithium levels of 0.23 to 0.50 mEq/L showed more robust and consistent reductions in brain free water, a neuroinflammation biomarker,^44,45,46^ than the 2 lithium-treated patients with levels less than 0.10 mEq/L.^24^

Although our PCC lithium dose-finding study results should be considered preliminary based on the very small number of patients treated in an open-label fashion, considering the large number of patients with neurologic PCC without any effective evidence-based treatments currently available, further clinical research on lithium therapy for treating neurologic PCC appears merited targeting serum lithium levels of approximately 0.18 to 0.50 mEq/L based on the totality of the data from our study.

### Limitations

In addition to the limitations involving small sample size and selection bias of the dose-finding study, a weakness of both the double-blind and dose-finding studies is the lack of any biomarker assessments. Biomarkers have the potential to enrich clinical trials by identifying patients most likely to benefit from a therapy and help bolster clinical outcome findings. As mentioned, neuroinflammation revealed by PET in several brain sites, including the thalamus, has shown promise as a neurologic PCC biomarker^7,8^; however, due to the increased cost and logistical challenges of PET, identification of MRI-assessed biomarkers would greatly facilitate their use in clinical trials. Recent MRI studies have shown disrupted blood-brain barrier and microstructural gray matter changes in several sites, including the thalamus, in patients with neurologic PCC compared with controls.^48,49,50^ Thalamic structural and microstructural changes have been shown to reflect cognitive dysfunction and fatigue in other neurologic conditions and, therefore, is a region of interest in neurologic PCC.^10,11,13^ PCC, Alzheimer disease, and Parkinson disease all share neuroinflammation as a mechanistic contributor and MRI-assessed free water, a measure of neuroinflammation, was shown to be more sensitive than other MRI assessments in distinguishing patients with Alzheimer and Parkinson disease from controls.^13,46,51,52^ These findings support future research assessing free water, particularly in the thalamus, as a potential neurologic PCC biomarker. Also, there is preliminary evidence that brain free water may be a druggable biomarker outcome responsive to brain anti-inflammatory therapies such as lithium.^24^

## Conclusions

In this RCT, therapy with lithium aspartate, 10 to 15 mg/d, was ineffective for treating PCC fatigue and cognitive dysfunction. Another RCT is required to assess the potential benefits of higher lithium dosages for treating neurologic PCC.

## Notes

Supplement 1.
Trial Protocol and Statistical Analysis Plan

Supplement 2.
Dose-Finding Study Protocol

Supplement 3.
Data Sharing Statement
