# Numeric Error in Abstract

**DOI:** 10.1001/jamanetworkopen.2024.44512

**Published:** 2024-10-24

In the Original Investigation titled “Lithium Aspartate for Long COVID Fatigue and Cognitive Dysfunction: A Randomized Clinical Trial,”^1^ published October 2, 2024, there was a numeric error in the Design, Setting, and Participants section of the Abstract. The phrase given as “less than 24” should have been “less than 29.” This article has been corrected.^1^
