# Supplementary material for: Lithium Aspartate for Long COVID Fatigue and Cognitive Dysfunction: A Randomized Clinical Trial
Source: JAMA Netw Open. 2024 Oct 2;7(10):e2436874. doi: 10.1001/jamanetworkopen.2024.36874 (PMC11447566; doi:10.1001/jamanetworkopen.2024.36874)
Supplement: Supplement 1. — Trial Protocol and Statistical Analysis Plan [file jamanetwopen-e2436874-s001.pdf]

University at Buffalo Institutional Review Board (UBIRB)

Office of Research Compliance | Clinical and Translational Research Center Room 5018
875 Ellicott St. | Buffalo, NY 14203
UB Federalwide Assurance ID#: FWA00008824

Complete Research Protocol (HRP-503)

Table of Contents

Template Instructions 2
1.0 Study Summary 4
2.0 Objectives* 4
3.0 Scientific Endpoints* 5
4.0 Background* 5
5.0 Study Design* 9
6.0 Study Intervention/Investigational Agent 9
7.0 Local Number of Subjects 10
8.0 Inclusion and Exclusion Criteria* 11
9.0 Vulnerable Populations* 12
10.0 Eligibility Screening* 14
11.0 Recruitment Methods 14
12.0 Procedures Involved* 15
13.0 Study Timelines* 18
14.0 Setting 18
15.0 Community-Based Participatory Research 19
16.0 Resources and Qualifications 19
17.0 Other Approvals 20
18.0 Provisions to Protect the Privacy Interests of Subjects 20
19.0 Data Management and Analysis* 21
20.0 Confidentiality* 21
A. Confidentiality of Study Data 21
B. Confidentiality of Study Specimens 22
21.0 Provisions to Monitor the Data to Ensure the Safety of Subjects* 23
22.0 Withdrawal of Subjects* 26
23.0 Risks to Subjects* 26
24.0 Potential Benefits to Subjects* 28
25.0 Compensation for Research-Related Injury 28
26.0 Economic Burden to Subjects 29
27.0 Compensation for Participation 29
28.0 Consent Process 29
29.0 Waiver or Alteration of Consent Process 34
30.0 Process to Document Consent 34
31.0 Multi-Site Research (Multisite/Multicenter Only)* 35
32.0 Banking Data or Specimens for Future Use* 36

Template Instructions

Sections that do not apply:

- *In several sections, the addition of checkboxes for **Not Applicable** have been added to the template as responses.*
    - *If an N/A checkbox is present, select the appropriate justification from the list.*
    - *If an N/A checkbox is not present, or if none of the existing checkboxes apply to your study, you must write in your own justification.*
- *In addition:*
    - *For research where the only study procedures are records/chart review: Sections 6, 21, 22, 24, 25, 26 and 27 do not apply.*
    - *For exempt research: Section 6 may not apply. Section 6.1 will still apply if there is a study intervention.*

Studies with multiple participant groups:

- *If this study involves multiple participant groups (e.g. parents and children), provide information in applicable sections for each participant group. Clearly label responses when they differ. For example:*

Response Example

Intervention Group: Lithium

Control Group: Placebo

Formatting:

- *Do not remove template instructions or section headings when they do not apply to your study.*

If you are pasting information from other documents using the "Merge Formatting" Paste option will maintain the formatting of the response boxes.

Amendments:

- *When making modifications or revisions to this and other documents, use the **Track Changes** function in Microsoft Word.*
- *Update the version date or number **on Page 3.***

PROTOCOL TITLE:

Include the full protocol title.

Response: Effect of low-dose lithium therapy on long COVID symptoms: a randomized controlled trial.

PRINCIPAL INVESTIGATOR:

Thomas Guttuso, Jr., MD; Department of Neurology; 716-932-6080; tguttuso@buffalo.edu

VERSION NUMBER/DATE:

Include the version number and date of this protocol.

Response: Version 5, 3/1/23

REVISION HISTORY

Revision #	Version Date	Summary of Changes	Consent Change?

FUNDING:

Indicate any funding for this proposal. This should match the Funding Sources page in Click IRB.

Response: Internal UB Department of Neurology funds and UL1TR001412.

GRANT APPLICABILITY:

Indicate whether this protocol is funded by a grant (e.g. NIH, foundation grant). For a grant with multiple aims, indicate which aims are covered by this research proposal.

NOTE: This question does not apply to studies funded by a sponsor contract.

Include a copy of the grant proposal with your submission.

Response: This study will be supported by the National Center for Advancing Translational Sciences of the National Institutes of Health under award number UL1TR001412 to the University at Buffalo.

RESEARCH REPOSITORY:

Indicate where the research files will be kept, including when the study has been closed. The repository should include, at minimum, copies of IRB correspondence (approval, determination letters) as well as signed consent documents. This documentation should be maintained for 3 years after the study has been closed.

Location: UBMD Neurology

Address: 5851 Main Street, Williamsville, NY 14221

Department: Neurology

1.0 Study Summary

**Study Title**	Effect of low-dose lithium therapy on long COVID symptoms: a randomized controlled trial.
**Study Design**	Randomized, parallel-group, double-blind, placebo-controlled trial.
**Primary Objective**	To determine if low-dose lithium can improve fatigue and/or brain fog in patients with long COVID.
**Secondary Objective(s)**	To determine if low-dose lithium provides a global improvement in symptoms, a higher level of well-being and improved quality of life.
**Research Intervention(s)/ Investigational Agent(s)**	Lithium
**IND/IDE #**	IND exempt per FDA Guidance
**Study Population**	Long COVID patients
**Sample Size**	50
**Study Duration for individual participants**	Five weeks
**Study Specific Abbreviations/ Definitions**	

2.0 Objectives*

2.1 Describe the purpose, specific aims, or objectives of this research.

Response: To determine if low-dose lithium reduces fatigue and/or brain fog and improves quality of life in patients with long COVID.

2.2 State the hypotheses to be tested, if applicable.

NOTE: A hypothesis is a specific, testable prediction about what you expect to happen in your study that corresponds with your above listed objectives.

Response: Low-dose lithium will reduce fatigue and brain fog and improve quality of life in patients with long COVID.

3.0 Scientific Endpoints*

3.1 Describe the scientific endpoint(s), the main result or occurrence under study.

NOTE: Scientific endpoints are outcomes defined before the study begins to determine whether the objectives of the study have been met and to draw conclusions from the data. Include primary and secondary endpoints. Some example endpoints are: reduction of symptoms, improvement in quality of life, or survival. Your response should ***not*** *be a date.*

Response:

Primary Endpoint:

Change from baseline to day 21 in combined Fatigue Severity Scale (FSS) and Brain Fog Severity Scale (BFSS) scores.

Secondary Endpoints:

Patient Global Impression of Change (PGIC) at day 21; Change from baseline to day 21 in Well-Being Scale scores; Desire to Continue Therapy Scale scores at day 21; Change from baseline to day 21 in Short Form-12 Health Survey scores (with 1-week modification); Change from baseline to day 21 in FSS scores in those with FSS score ≥28 at baseline; Change from baseline to day 21 in BFSS scores in those with BFSS score ≥28 at baseline; Change from baseline to day 21 in Generalized Anxiety Disorder Scale scores in those with score ≥1 at baseline; Change from baseline to day 21 in Headache and Body Pain Bother Scale scores in those with score ≥2 at baseline; Change from baseline to day 21 in Insomnia Severity Index scores in those with score ≥10 at baseline; Sense of Smell and Taste Change Scale scores at day 21 in those with a Sense of Smell and Taste Impairment Scale score ≥2 at baseline; Change from baseline to day 21 in Digit Symbol Substitution Test scores; Change from baseline to day 21 in immediate and delayed word recall test scores.

4.0 Background*

4.1 Provide the scientific or scholarly background, rationale, and significance of the research based on the existing literature and how it will contribute to existing knowledge. Describe any gaps in current knowledge. Include relevant preliminary findings or prior research by the investigator.

Response:

Long COVID, also known as long-haul COVID or post-acute sequelae of SARS CoV-2 infection (PASC), is defined by the Centers for Disease Control and Prevention (CDC) as having persistent symptoms for >4 weeks after recovering from the infectious phase of COVID-19. The most common long COVID symptoms are fatigue, concentration/cognitive impairment (also called “brain fog”) and shortness of breath with exertion. In addition, other long COVID symptoms include dizziness when standing, chronic headaches, diffuse muscle and/or joint pains, abdominal pain, decreased sense of smell or taste, coordination problems, insomnia, anxiety and depression.[1] The long

COVID symptoms stemming from the brain- such as fatigue, brain fog, headaches, etc.- have been coined “neuro long COVID” symptoms. It is estimated that about 20-30% of people infected with COVID develop long COVID with varying degrees of severity.[2] About 30 million people in the US are estimated to have long COVID and over one million are currently disabled from it.

Although the cause of neuro long COVID is currently unknown, evidence supports increased neuroinflammation as a contributor to the pathophysiology perhaps being triggered by COVID viral proteins persisting in brain cells.[3] A recent study showed significantly increased inflammatory markers of C-reactive protein (CRP) and serum amyloid A in the cerebrospinal fluid (CSF) of long COVID patients with brain fog compared to post-COVID patients without any long COVID.[4] In addition, there were trends towards increased CSF levels of several other inflammatory markers in neuro long COVID patients. There is also evidence that COVID-19-induced inflammation may reactivate the Epstein-Barr Virus (EBV) in long COVID patients and contribute to the symptom similarities between patients with long COVID and those with EBV reactivation.[5]

However, it does not appear that increased brain inflammation is reflected in the systemic circulation of long COVID patients. One study did report significantly increased blood acute phase reactants; such as erythrocyte sedimentation rate (ESR), c-reactive protein (CRP), D-dimer and ferritin; among 120 patients who had tested positive for COVID-19 about 4 months previously compared to 120 matched control patients; however, this study did not assess patients for long COVID symptoms.[6] A more recent study enrolled 189 patients between July 2020 and July 2021 who had tested positive for COVID-19 about 5 months previously including 104 patients with long COVID.[7] This study found no significant differences between either the whole COVID-19 cohort or the long COVID subgroup and 120 control patients who never had COVID for blood CRP, D-dimer or plasma neurofilament light or detection of antinuclear antibody (ANA), anticardiolipin antibody or rheumatoid factor antibody. Another study reported an increase in several proinflammatory cytokines in the serum of post-COVID patients compared to controls but with no differences observed between long COVID and post-COVID patients without long COVID symptoms.[8] Thus, the current body of evidence has yet to identify any serum biomarkers for long COVID.

Because increased brain inflammation may be involved in the pathophysiology of neuro long COVID and microglia are the primary immune cell in the brain mediating neuroinflammation, therapies known to cross the blood-brain barrier (BBB) and suppress microglial activation could potentially be effective for treating patients with neuro long COVID.

Lithium has been shown to effectively suppress microglial activation and reduce serum inflammatory cytokines including interleukin-6 (IL-6) and tumor necrosis factor-alpha (TNF-a).[9-12] Lithium is well known to be centrally active as a highly effective treatment for bipolar disorder and readily crosses the BBB and cell membranes via passage through sodium channels.[13,14] Although the elemental lithium dosage required to treat bipolar disorder is about 300mg/day, there is clinical evidence that dosages <1mg/day may prevent and slow Alzheimer’s disease (AD) and Parkinson’s disease (PD).[15-17] Chronic brain inflammation has been implicated in the pathophysiology of both AD and PD.[18]

The term “elemental lithium” refers to the weight of lithium alone not including the weight of the salt carrier bound to lithium such as the carbonate moiety in prescription lithium carbonate or the aspartate or orotate moieties in over-the-counter (OTC) lithium dietary supplement products. The OTC lithium dietary supplement products available are lithium aspartate and lithium orotate, which typically provide 5mg of elemental

lithium/capsule. Because orotate has been shown to increase the incidences of several cancers in rodent cancer models,[19,20] we chose to study the effects of lithium aspartate on blood-based and MRI biomarkers in a recent pilot PD clinical trial. In PD patients receiving about 150mg/day of elemental lithium as lithium carbonate or 45mg/day or 15mg/day of elemental lithium as lithium aspartate for six months, mean trough serum lithium levels were 1282, 828 and 195µg/L, respectively, compared to 0.73µg/L in PD patients not receiving lithium (*unpublished data*). These data confirm that OTC lithium aspartate contains bioavailable elemental lithium.

Preliminary data:

Because neuroinflammation may be involved in long COVID pathophysiology and low-dose lithium may have neuroprotective effects in neurodegenerative diseases that are known to have increased neuroinflammation, lithium aspartate 5mg/day was suggested by Dr. Guttuso in March 2022 for use in a 17-year-old male with long COVID. This patient had severe fatigue, brain fog and daily headaches for 10 weeks after testing positive for COVID-19 and had failed therapy with prednisone and baclofen for these symptoms. These symptoms were disabling as the patient was unable to attend high school or lacrosse practices and spent most of the day lying in bed, according to his parents. Within two days of starting lithium aspartate 5mg/day, the patient reported about 90% improvements in all three of his neuro long COVID symptoms and was able to return to classes. Three weeks later he developed another viral infection and tested negative for COVID but had recurrences of his neuro long COVID symptoms. He increased lithium aspartate to 10mg/day and again reported about 90% improvements in these symptoms within 3-5 days. Encouraged by this anecdotal case, lithium aspartate has been recommended to eight additional patients with neuro long COVID symptoms for at least six weeks after testing positive for COVID-19. Most but not all of these patients have reported some degree of benefit to their long COVID symptoms after starting low-dose lithium therapy. Anecdotally, it appeared that the symptoms of fatigue and brain fog improved to the greatest extent. No side effects have been reported by any patient that could potentially be from lithium. These preliminary reports have justified conducting this proposed research study to formally determine whether or not low-dose lithium therapy can reduce symptoms of long COVID.

4.2 Include complete citations or references.

Response:

1. Taquet M, Dercon Q, Luciano S, Geddes JR, Husain M, Harrison PJ. Incidence, co-occurrence, and evolution of long-COVID features: A 6-month retrospective cohort study of 273,618 survivors of COVID-19. PLoS Med 2021;18:e1003773. doi: 10.1371/journal.pmed.1003773

2. Nehme M, Braillard O, Alcoba G, Aebischer Perone S, Courvoisier D, Chappuis F, Guessous I, Covicare T. COVID-19 Symptoms: Longitudinal Evolution and Persistence in Outpatient Settings. Ann Intern Med 2021;174:723-5. doi: 10.7326/M20-5926

3. Peluso MJ, Deeks SG, Mustapic M, Kapogiannis D, Henrich TJ, Lu S, Goldberg SA, Hoh R, Chen JY, Martinez EO, Kelly JD, Martin JN, Goetzl EJ. SARS-CoV-2 and

Mitochondrial Proteins in Neural-Derived Exosomes of COVID-19. Ann Neurol 2022;91:772-81. doi: 10.1002/ana.26350
4. Medpagetoday. Post-COVID Brain Fog Tied to CSF Markers- Overstimulated immune system may be impetus for long COVID cognitive symptoms. Available at: https://www.medpagetoday.com/meetingcoverage/aanvideopearls/98559 Accessed 20 July 2022. doi:
5. Gold JE, Okyay RA, Licht WE, Hurley DJ. Investigation of Long COVID Prevalence and Its Relationship to Epstein-Barr Virus Reactivation. Pathogens 2021;10. doi: 10.3390/pathogens10060763
6. Gameil MA, Marzouk RE, Elsebaie AH, Rozaik SE. Long-term clinical and biochemical residue after COVID-19 recovery. Egypt Liver J 2021;11:74. doi: 10.1186/s43066-021-00144-1
7. Sneller MC, Liang CJ, Marques AR, Chung JY, Shanbhag SM, Fontana JR, Raza H, Okeke O, Dewar RL, Higgins BP, Tolstenko K, Kwan RW, Gittens KR, Seamon CA, McCormack G, Shaw JS, Okpali GM, Law M, Trihemasava K, Kennedy BD, Shi V, Justement JS, Buckner CM, Blazkova J, Moir S, Chun TW, Lane HC. A Longitudinal Study of COVID-19 Sequelae and Immunity: Baseline Findings. Ann Intern Med 2022. doi: 10.7326/M21-4905
8. Phetsouphanh C, Darley DR, Wilson DB, Howe A, Munier CML, Patel SK, Juno JA, Burrell LM, Kent SJ, Dore GJ, Kelleher AD, Matthews GV. Immunological dysfunction persists for 8 months following initial mild-to-moderate SARS-CoV-2 infection. Nat Immunol 2022;23:210-6. doi: 10.1038/s41590-021-01113-x
9. Dong H, Zhang X, Dai X, Lu S, Gui B, Jin W, Zhang S, Zhang S, Qian Y. Lithium ameliorates lipopolysaccharide-induced microglial activation via inhibition of toll-like receptor 4 expression by activating the PI3K/Akt/FoxO1 pathway. Journal of neuroinflammation 2014;11:140. doi: 10.1186/s12974-014-0140-4
10. Yu F, Wang Z, Tchantchou F, Chiu CT, Zhang Y, Chuang DM. Lithium ameliorates neurodegeneration, suppresses neuroinflammation, and improves behavioral performance in a mouse model of traumatic brain injury. J Neurotrauma 2012;29:362-74. doi: 10.1089/neu.2011.1942
11. Valvassori SS, Tonin PT, Varela RB, Carvalho AF, Mariot E, Amboni RT, Bianchini G, Andersen ML, Quevedo J. Lithium modulates the production of peripheral and cerebral cytokines in an animal model of mania induced by dextroamphetamine. Bipolar Disord 2015;17:507-17. doi: 10.1111/bdi.12299
12. Spuch C, Lopez-Garcia M, Rivera-Baltanas T, Cabrera-Alvargonzalez JJ, Gadh S, Rodrigues-Amorim D, Alvarez-Estevez T, Mora A, Iglesias-Martinez-Almeida M, Freiria-Martinez L, Perez-Rodriguez M, Perez-Gonzalez A, Lopez-Dominguez A, Longueira-Suarez MR, Sousa-Dominguez A, Araujo-Ameijeiras A, Mosquera-Rodriguez D, Crespo M, Vila-Fernandez D, Regueiro B, Olivares JM. Efficacy and Safety of Lithium Treatment in SARS-CoV-2 Infected Patients. Front Pharmacol 2022;13:850583. doi: 10.3389/fphar.2022.850583
13. Janka Z, Jones DG. Lithium entry into neural cells via sodium channels: a morphometric approach. Neuroscience 1982;7:2849-57. doi:
14. Lahteenvuo M, Tanskanen A, Taipale H, Hoti F, Vattulainen P, Vieta E, Tiihonen J. Real-world Effectiveness of Pharmacologic Treatments for the Prevention of Rehospitalization in a Finnish Nationwide Cohort of Patients With Bipolar Disorder. JAMA Psychiatry 2018;75:347-55. doi: 10.1001/jamapsychiatry.2017.4711

15. Guttuso T, Jr. High lithium levels in tobacco may account for reduced incidences of both Parkinson's disease and melanoma in smokers through enhanced β-catenin mediated activity. Med Hypothesis 2019;131. doi:
16. Kessing LV, Gerds TA, Knudsen NN, Jorgensen LF, Kristiansen SM, Voutchkova D, Ernstsen V, Schullehner J, Hansen B, Andersen PK, Ersboll AK. Association of Lithium in Drinking Water With the Incidence of Dementia. JAMA Psychiatry 2017;74:1005-10. doi: 10.1001/jamapsychiatry.2017.2362
17. Nunes MA, Viel TA, Buck HS. Microdose lithium treatment stabilized cognitive impairment in patients with Alzheimer's disease. Curr Alzheimer Res 2013;10:104-7. doi:
18. Guzman-Martinez L, Maccioni RB, Andrade V, Navarrete LP, Pastor MG, Ramos-Escobar N. Neuroinflammation as a Common Feature of Neurodegenerative Disorders. Front Pharmacol 2019;10:1008. doi: 10.3389/fphar.2019.01008
19. Laconi E, Denda A, Rao PM, Rajalakshmi S, Pani P, Sarma DS. Studies on liver tumor promotion in the rat by orotic acid: dose and minimum exposure time required for dietary orotic acid to promote hepatocarcinogenesis. Carcinogenesis 1993;14:1771-5. doi:
20. Laconi E, Vasudevan S, Rao PM, Rajalakshmi S, Pani P, Sarma DS. The effect of long-term feeding of orotic acid on the incidence of foci of enzyme-altered hepatocytes and hepatic nodules in Fischer 344 rats. Carcinogenesis 1993;14:1901-5. doi: 10.1093/carcin/14.9.1901
21. Lerdal A, Johansson S, Kottorp A, von Koch L. Psychometric properties of the Fatigue Severity Scale: Rasch analyses of responses in a Norwegian and a Swedish MS cohort. Mult Scler 2010;16:733-41. doi: 10.1177/1352458510370792
22. Lerdal A, Kottorp A. Psychometric properties of the Fatigue Severity Scale-Rasch analyses of individual responses in a Norwegian stroke cohort. Int J Nurs Stud 2011;48:1258-65. doi: 10.1016/j.ijnurstu.2011.02.019
23. Lerdal A, Kottorp A, Gay C, Aouizerat BE, Portillo CJ, Lee KA. A 7-item version of the fatigue severity scale has better psychometric properties among HIV-infected adults: an application of a Rasch model. Qual Life Res 2011;20:1447-56. doi: 10.1007/s11136-011-9877-8
24. Ware J, Jr., Kosinski M, Keller SD. A 12-Item Short-Form Health Survey: construction of scales and preliminary tests of reliability and validity. Med Care 1996;34:220-33. doi: 10.1097/00005650-199603000-00003

5.0 Study Design*

5.1 Describe and explain the study design (e.g. case-control, cross-sectional, ethnographic, experimental, interventional, longitudinal, observational).

Response:

Randomized, double-blind, placebo-controlled, parallel-design trial.

6.0 Study Intervention/Investigational Agent

6.1 Describe the study intervention and/or investigational agent (e.g., drug, device) that is being evaluated.

Response:

Lithium aspartate

6.2 Drug/Device Handling: If the research involves drugs or device, describe your plans to store, handle, and administer those drugs or devices so that they will be used only on subjects and be used only by authorized investigators.

- *If the control of the drugs or devices used in this protocol will be accomplished by following an established, approved organizational SOP (e.g., Research Pharmacy SOP for the Control of Investigational Drugs, etc.), please reference that SOP in this section.*

Response:

Solaray lithium aspartate 5mg capsules manufactured by Nutraceutical Corporation (Salt Lake City, UT) will be used for this study. Identically-appearing placebo capsules will be manufactured by UB's CTSA research pharmacy. All study capsules will be over-encapsulated with size 0 enteric protected capsules (Capsuline, Pompano Beach, FL) to prevent ingredient breakdown in the stomach.

6.3 If the drug is investigational (has an IND) or the device has an IDE or a claim of abbreviated IDE (non-significant risk device), include the following information:

- *Identify the holder of the IND/IDE/Abbreviated IDE.*
- *Explain procedures followed to comply with sponsor requirements for FDA regulated research for the following:*

	Applicable to:		
FDA Regulation	***IND Studies***	***IDE studies***	***Abbreviated IDE studies***
21 CFR 11	***X***	***X***	
21 CFR 54	***X***	***X***	
21 CFR 210	***X***		
21 CFR 211	***X***		
21 CFR 312	***X***		
21 CFR 812		***X***	***X***
21 CFR 820		***X***	

Response:

7.0 Local Number of Subjects

7.1 Indicate the total number of subjects that will be enrolled or records that will be reviewed locally.

Response:

50

7.2 *If applicable, indicate how many subjects you expect to screen to reach your target sample (i.e. your screen failure rate).*

Response:

200

7.3 *Justify the feasibility of recruiting the proposed number of eligible subjects within the anticipated recruitment period. For example, how many potential subjects do you have access to? What percentage of those potential subjects do you need to recruit?*

Response:

Patients will be recruited through advertisements and through local primary care offices. Based on the prevalence of long COVID, there are likely about 50,000 people in Western New York with long COVID who may be eligible for this trial.

8.0 Inclusion and Exclusion Criteria*

8.1 *Describe the criteria that define who will be* ***included*** *in your final study sample.*

NOTE: This may be done in bullet point fashion.

Response:

Patient Eligibility Criteria (Inclusion and Exclusion Criteria):

1) Documented or self-reported positive test for COVID-19 ≥4 weeks prior to enrollment.
2) No fever for ≥4 weeks prior to enrollment.
3) No COVID vaccine for ≥4 weeks prior to enrollment and not planning on obtaining a COVID vaccine within the next 5 weeks.
4) Is 18-80 years old.
5) Reports fatigue and/or brain fog (i.e. concentration or memory impairment) for ≥4 weeks prior to enrollment on the PASC-Specific Symptom questionnaire beginning after infection with COVID-19.
6) Fatigue Severity Scale (FSS) score ≥28 or Brain Fog Severity Scale (BFSS) score ≥28 at baseline.
7) Beck Depression Inventory II score ≤28.
8) No change in any psychoactive or steroid medications for ≥30 days.
9) No plan to change any psychoactive, steroid or diuretic medication for ≥5 weeks.
10) Not using any long COVID therapies felt to be worsening the patient’s symptoms or starting a long COVID therapy within the next 5 weeks.
11) No history of fibromyalgia, chronic fatigue syndrome, chronic kidney disease, rheumatoid arthritis, thyroid function abnormalities or progressive cognitive disorder prior to COVID-19 infection.
12) No history of heart attack or stroke within the previous year.
13) No active medical, psychiatric or social problems that would interfere with completing the study procedures in the opinion of the investigator.
14) No use of lithium, tobacco or marijuana products for ≥30 days and no current alcohol abuse (≥4 drinks/day) or illicit drug use. No daily NSAID use.
15) Not receiving or applying for disability payments or workman’s compensation for long COVID.

16) Not pregnant or nursing or planning to get pregnant over the next two months.

8.2 *Describe the criteria that define who will be* ***excluded*** *from your final study sample.*

NOTE: This may be done in bullet point fashion.

Response:

See 8.1.

8.3 *Indicate specifically whether you will include any of the following special populations in your study using the checkboxes below.*

NOTE: Members of special populations may not be targeted for enrollment in your study unless you indicate this in your inclusion criteria.

Response:

☐ Adults unable to consent

☐ Individuals who are not yet adults (infants, children, teenagers)

☐ Pregnant women

☐ Prisoners

8.4 *Indicate whether you will include non-English speaking individuals in your study.* ***Provide justification if you will exclude non-English speaking individuals.***

In order to meet one of the primary ethical principles of equitable selection of subjects, non-English speaking individuals may ***not*** *be routinely excluded from research as a matter of convenience.*

In cases where the research is of therapeutic intent or is designed to investigate areas that would necessarily require certain populations who may not speak English, the researcher is required to make efforts to recruit and include non-English speaking individuals. However, there are studies in which it would be reasonable to limit subjects to those who speak English. Some examples include pilot studies, small unfunded studies with validated instruments not available in other languages, studies with numerous questionnaires, and some non-therapeutic studies which offer no direct benefit.

Response:

Only English-speaking individuals will be enrolled in this study as many of the study questionnaires are only available in English.

9.0 Vulnerable Populations*

If the research involves special populations that are considered vulnerable, ***describe the safeguards included to protect their rights and welfare.***

NOTE: You should refer to the appropriate checklists, referenced below, to ensure you have provided adequate detail regarding safeguards and protections. You do not, however, need to provide these checklists to the IRB.

9.1 For research that involves ***pregnant women,*** *safeguards include:*
NOTE CHECKLIST: Pregnant Women (HRP-412)

Response:

☒ **N/A**: This research does not involve pregnant women.

9.2 For research that involves ***neonates of uncertain viability or non-viable neonates,*** *safeguards include:*
NOTE CHECKLISTS: Non-Viable Neonates (HRP-413), or Neonates of Uncertain Viability (HRP-414)

Response:

☒ **N/A:** This research does not involve non-viable neonates or neonates of uncertain viability.

9.3 For research that involves ***prisoners****, safeguards include:*
NOTE CHECKLIST: Prisoners (HRP-415)

Response:

☒ **N/A:** This research does not involve prisoners.

9.4 For research that involves ***persons who have not attained the legal age for consent to treatments or procedures involved in the research ("children")****, safeguards include:*
NOTE CHECKLIST: Children (HRP-416)

Response:

☒ **N/A:** This research does not involve persons who have not attained the legal age for consent to treatments or procedures ("children").

9.5 For research that involves ***cognitively impaired adults****, safeguards include:*
NOTE CHECKLIST: Cognitively Impaired Adults (HRP-417)

Response:

☒ **N/A:** This research does not involve cognitively impaired adults.

9.6 Consider if other specifically targeted populations such as students, employees of a specific firm, or educationally or economically disadvantaged persons are vulnerable. ***Provide information regarding***

their safeguards and protections, including safeguards to eliminate coercion or undue influence.

Response:

10.0 Eligibility Screening*

10.1 Describe **screening procedures** *for determining subjects' eligibility. Screening refers to determining if prospective participants meet inclusion and exclusion criteria.*

Include all relevant screening documents with your submission (e.g. screening protocol, script, questionnaire).

Response:

People who call the study line for information on the study will be asked the questions on the Long COVID Study Patient Screening Form.

☐ **N/A:** There is no screening as part of this protocol.

11.0 Recruitment Methods

☐ **N/A:** This is a records review only, and subjects will not be recruited. NOTE: If you select this option, please make sure that all records review procedures and inclusion/exclusion screening are adequately described in other sections.

11.1 Describe when, where, and how potential subjects will be recruited.

NOTE: Recruitment refers to how you are identifying potential participants and introducing them to the study. Include specific methods you will use (e.g. searching charts for specific ICD code numbers, Research Participant Groups, posted advertisements, etc.).

Response:

A study advertisement will be placed in the Buffalo News and Bee Newspapers and their on-line sites. In addition, local primary care offices will be provided with the study advertisement to post in their office and provide to any of their long COVID patients that they think may be interested in the study.

The Clinical and Translational Science Institute's (CTSI) Community Engagement Team (CET) hosts the Buffalo Research Registry (BRR, IRB Approved STUDY00000806), a resource that connects researchers looking to recruit participants and community members looking to get involved with research. To participate in the registry, volunteers complete a voluntary intake form. Volunteers have agreed to be contacted about potential research opportunities based on their self-reported information.

As described in the BRR's IRB Approved Protocol (STUDY00000806), key personnel from the CTSI will serve as the conduit between our research team and the registry volunteers. Key personnel will verify our IRB study approval, review inclusion and

exclusion criteria and will sort the registry data accordingly.

In terms of recruiting for our study, key personnel will pull volunteer reports using the inclusion and exclusion criteria. Key personnel will centrally invite volunteers to participate in the study and provide a warm hand off to our team. There are two ways we are sharing our study. These include:

Electronically- Using the volunteer report from REDCap, key personnel from the CTSI will send initial e-mail invites to introduce our study to registry volunteers.

Post Mail- Key personnel from the CTSI will provide our team with contact information (i.e. first name, last name, post mail address) to prepare a mailing. This information will be shared in a password protected excel spread sheet. The password to the spreadsheet will be sent in a separate follow-up e-mail. We will only use the list for this protocol and we agree to destroy the list once recruitment for the study has closed. We also plan on conducting follow-up by phone/email after initial contact. This contact information will be provided in a password protected excel spread sheet. The password to the spreadsheet will be sent in a separate follow-up e-mail. We will only use the list for this protocol and we agree to destroy the list once recruitment for the study has closed.

We will have no access to the health information provided in the registry.

11.2 Describe how you will protect the privacy interests of prospective subjects during the recruitment process.

NOTE: Privacy refers to an individual's right to control access to him or herself.

Response: Potential participants will respond to study ads at times and places where they control their own privacy. When discussing the study in the office, the PI will talk with potential participants in a private room.

11.3 Identify any materials that will be used to recruit subjects.

NOTE: Examples include scripts for telephone calls, in person announcements / presentations, email invitations.

For advertisements, include the final copy of printed advertisements with your submission. When advertisements are taped for broadcast, attach the final audio/video tape. NOTE: You may submit the wording of the advertisement prior to taping to ensure there will be no IRB-required revisions, provided the IRB also reviews and approves the final version.

Response:

The study advertisement is included in this submission.

12.0 Procedures Involved*

*12.1 Provide a description of **all research procedures or activities** being performed and when they are performed once a subject is screened and determined to be eligible. Provide as much detail as possible.*

NOTE: This should serve as a blueprint for your study and include enough detail so that another investigator could pick up your protocol and replicate the research. For studies that have multiple or complex visits or procedures, consider the addition of a schedule of events table in in your response.

Response:

Baseline Visit (Visit 1)– performed about 21 days before Visit 2

- Personal Information (name, date of birth, gender, race/ethnicity, etc.).
- Medical History focusing on your long COVID symptoms assessed by the PASC-Specific Symptom questionnaire.
- Review of symptoms and medications taken.
- Completion of the following questionnaires: The Headache and Body Pain Bother Scale, the Generalized Anxiety Disorder Scale, the Digit Symbol Substitution Test, the Short Form-12 Health Survey, the Fatigue Severity Scale, the Well Being Scale, the Sense of Smell and Taste Impairment Scale, the Brain Fog Severity Scale, the Beck Depression Inventory-II, and the Insomnia Severity Index.
- This visit will take about 90 minutes.

Women of child-bearing potential will need to have a negative urine pregnancy test at Visit 1 to be eligible.

Eligible patients will be randomly assigned to receive either lithium or identically-appearing placebo capsules (1:1) in a double-blind fashion. A blocked randomization schedule will be devised by the study's statistician, Greg Wilding, PhD. Study drug will be coded by the CTSA research pharmacy to comply with the randomization schedule and ensure all study personnel remain blinded.

Patients will take two study capsules (either 10mg total of lithium or matching placebo capsules) every morning. If after 10 days, a patient is still bothered by any long COVID symptoms, the dosage can be increased to three study capsules every morning for the last 11 days before returning to the clinic for the "End of Double-Blind Study Visit".

Patients will be instructed to contact the study team if they experience any side effects or have any questions.

End of Double-Blind Study Visit (Visit 2)– performed about 21 days after Visit 1

- Patients will be instructed to bring any unused study capsules to this visit for drug accountability.
- Patients will be asked about any side effects that they may have experienced during the study.
- Patients will complete the same questionnaires completed during Visit 1 in addition to the Patient Global Impression of Change (PGIC) scale, the Change in Sense of Smell and Taste Scale, and the Desire to Continue Therapy Scale.
- This visit will take about 60 minutes.

This will end the double-blind phase of the study. For patients who have completed Visits 1 and 2, they will then be offered lithium capsules to take for two additional weeks without the chance of receiving placebo capsules. Patients will start with two lithium

aspartate 5mg capsules every morning. If they are still bothered by long COVID symptoms after a week, the daily dosage can be increased to three lithium capsules every morning for the last week. At the end of the second week of lithium treatment, patients will be asked to complete the provided questionnaires from Visit 2 excluding the Digit Symbol Substitution Test and mail them to the study team in a provided self-addressed stamped envelope. This will end the study.

12.2 Describe what data will be collected.

NOTE: For studies with multiple data collection points or long-term follow up, consider the addition of a schedule or table in your response.

Response:

Patient medical history and demographic information. Questionnaires.

12.3 List any instruments or measurement tools used to collect data (e.g. questionnaire, interview guide, validated instrument, data collection form).

Include copies of these documents with your submission.

Response:

The Headache and Body Pain Bother Scale, the Generalized Anxiety Disorder Scale (GAD-2), the Short Form-12 Health Survey (SF-12 quality of life survey), the Fatigue Severity Scale, the Well Being Scale, the Sense of Smell and Taste Impairment Scale, the Brain Fog Severity Scale, the Beck Depression Inventory-II (BDI-II), the Insomnia Severity Index, the Patient Global Impression of Change (PGIC) scale, the Change in Sense of Smell and Taste Scale, the Digit Symbol Substitution Test and the Desire to Continue Therapy Scale.

12.4 Describe any source records that will be used to collect data about subjects (e.g. school records, electronic medical records).

Response: Patients' electronic medical records assessed through Cerner and Allscripts and new health information obtained through this research study including questionnaire data will be used.

12.5 Indicate whether or not ***individual*** *subject results, such as results of investigational diagnostic tests, genetic tests, or incidental findings will be shared with subjects or others (e.g., the subject's primary care physician) and if so, describe how these will be shared.*

Response:

Individual patients will be informed of their treatment allocation by mail after the study is completed.

12.6 Indicate whether or not ***study*** *results will be shared with subjects or others, and if so, describe how these will be shared.*

Response: Individual patients will be not be informed of the study results. The final results from this study will be presented in medical meetings and published in a medical journal.

13.0 Study Timelines*

13.1 Describe the anticipated duration needed to enroll all study subjects.

Response:

11 months

13.2 Describe the duration of an individual subject's participation in the study. Include length of study visits, and overall study follow-up time.

Response:

Visit 1 will take about 90 minutes and Visit 2 about 60 minutes. Each patient will be in the study for five weeks.

13.3 Describe the estimated duration for the investigators to complete this study (i.e. all data is collected and all analyses have been completed).

Response:

10 months

14.0 Setting

14.1 Describe all facilities/sites where you will be conducting research procedures. Include a description of the security and privacy of the facilities (e.g. locked facility, limited access, privacy barriers). Facility, department, and type of room are relevant. Do not abbreviate facility names.

NOTE: Examples of acceptable response may be: "A classroom setting in the Department of Psychology equipped with a computer with relevant survey administration software," "The angiogram suite at Buffalo General Medical Center, a fully accredited tertiary care institution within New York State with badge access," or, "Community Center meeting hall."

Response:

All clinical visits will occur at UBMD Neurology at 5851 Main Street, Williamsville, NY 14221. This is a clinic where neurology patients are routinely treated. The facility is staffed during business hours and otherwise locked.

14.2 For research conducted outside of UB and its affiliates, describe:

- *Site-specific regulations or customs affecting the research*
- *Local scientific and ethical review structure*

NOTE: This question is referring to UB affiliated research taking place outside UB, i.e. research conducted in the community, school-based research, international research, etc. It is not referring to multi-site research. UB affiliated institutions include Kaleida Health, ECMC, and Roswell Park Cancer Institute.

Response:

☒ **N/A:** This study is not conducted outside of UB or its affiliates.

15.0 Community-Based Participatory Research

15.1 Describe involvement of the community in the design and conduct of the research.

NOTE: Community-Based Participatory Research (CBPR) is a collaborative approach to research that equitably involves all partners in the research process and recognizes the unique strengths that each brings. CBPR begins with a research topic of importance to the community, has the aim of combining knowledge with action and achieving social change to improve health outcomes and eliminate health disparities.

Response:

☒ **N/A:** This study does not utilize CBPR.

15.2 Describe the composition and involvement of a community advisory board.

Response:

☒ **N/A:** This study does not have a community advisory board.

16.0 Resources and Qualifications

*16.1 Describe the qualifications (e.g., education, training, experience, expertise, or certifications) of the Principal Investigator **and** staff to perform the research. When applicable describe their knowledge of the local study sites, culture, and society. Provide enough information to convince the IRB that you have qualified staff for the proposed research.*

NOTE: If you specify a person by name, a change to that person will require prior approval by the IRB. If you specify a person by role (e.g., coordinator, research assistant, co-investigator, or pharmacist), a change to that person will not usually require prior approval by the IRB, provided that the person meets the qualifications described to fulfill their roles.

Response:

The PI, Thomas Guttuso, Jr., MD is a Professor of Neurology at UB. Dr. Guttuso has served as PI on nine, hypothesis-driven clinical trials and site-PI on three, industry-sponsored clinical trials. The study coordinator is a nurse practitioner with over 10 years of experience in this role on clinical trials.

Describe other resources available to conduct the research.

16.2 Describe the time and effort that the Principal Investigator and research staff will devote to conducting and completing the research.

NOTE: Examples include the percentage of Full Time Equivalents (FTE), hours per week. The question will elicit whether there are appropriate resources to conduct the research.

Response:

The PI will devote 0.2FTE and the coordinator 0.2FTE towards this project.

16.3 Describe the availability of medical or psychological resources that subjects might need as a result of anticipated consequences of the human research, if applicable.

NOTE: One example includes: on-call availability of a counselor or psychologist for a study that screens subjects for depression.

Response: N/A

16.4 Describe your process to ensure that all persons assisting with the research are adequately informed about the protocol, the research procedures, and their duties and functions.

Response:

The PI will directly communicate with all study staff to review study procedures and define each member's duties. The PI will monitor all staff throughout the study to ensure that study procedures are being followed appropriately.

17.0 Other Approvals

17.1 Describe any approvals that will be obtained prior to commencing the research (e.g., school, external site, funding agency, laboratory, radiation safety, or biosafety).

Response:

☒ **N/A:** This study does not require any other approvals.

18.0 Provisions to Protect the Privacy Interests of Subjects

18.1 Describe how you will protect subjects' privacy interests during the course of this research.

NOTE: Privacy refers to an individual's right to control access to him or herself. Privacy applies to the person. Confidentiality refers to how data collected about individuals for the research will be protected by the researcher from release. Confidentiality applies to the data.

Examples of appropriate responses include: "participant only meets with a study coordinator in a classroom setting where no one can overhear", or "the participant is reminded that they are free to refuse to answer any questions that they do not feel comfortable answering."

Response:

Study subjects will meet with either the PI or coordinator in a clinic room with the door shut for privacy.

18.2 Indicate how the research team is permitted to access any sources of information about the subjects.

NOTE: Examples of appropriate responses include: school permission for review of records, consent of the subject, HIPAA waiver. This question ***does apply*** *to records reviews.*

Response:

Consent of the subject.

19.0 Data Management and Analysis*

19.1 Describe the data analysis plan, including any statistical procedures. This section applies to both quantitative and qualitative analysis.

Response:

To describe the observed variability in the data and test for differences between groups defined by randomized assignment, a linear model will be fit to each considered outcome. Specifically, each numeric outcome will be modeled as a function of randomized group assignment and the baseline value of the outcome variable. Once the model is fit, a linear contrast based on the estimated model parameters will be constructed and used to test for the overall effect of random assignment using an approximate F-test as implemented by SAS PROC MIXED. A point estimate and corresponding confidence interval to quantify treatment differences will be provided. All tests will be two-sided and tested at a 0.05 nominal significance level. Standard diagnostic plots will be used to assess model fit and transformations of variables may be considered in order to meet statistical assumptions.

19.2 If applicable, provide a power analysis.

NOTE: This may not apply to certain types of studies, including chart/records reviews, survey studies, or observational studies. This question is asked to elicit whether the investigator has an adequate sample size to achieve the study objectives and justify a conclusion.

Response:

Assuming a 48% standard deviation in the Primary Endpoint in the placebo group and a 40% treatment effect size, a total of 50 patients will be needed to detect an intergroup difference with 80% power at a 2-tailed alpha of 0.05.

19.3 Describe any procedures that will be used for quality control of collected data.

Response:

After patients complete the questionnaires, the PI will review each to confirm that all questions were answered.

20.0 Confidentiality*

A. Confidentiality of Study Data

Describe the local procedures for maintenance of confidentiality of ***study data and any records that will be reviewed for data collection****.*

20.1 A. Where and how will all data and records be stored? Include information about: password protection, encryption, physical controls, authorization of access, and separation of identifiers and data, as applicable. Include physical (e.g. paper) ***and*** *electronic files.*

Response:

A study binder will be assembled for each subject that will contain all of their study data. However, the forms linking subjects' personal identifiable information to their study ID numbers will be kept in a separate folder. This folder and all study binders will be stored in a locked room in a locked cabinet at the study site. These data will be entered into an excel file in a password protected computer located in this same locked room. The excel file will only contain subject ID numbers and no personal identifiable information.

20.2 A. How long will the data be stored?

Response:

IRB correspondences and signed consent forms will be kept for at least three years. Patient identifiers will be deleted at the first available opportunity after it is no longer necessary to conduct the research. Data analysis files that contain no dates or patient identifiers will be kept indefinitely.

20.3 A. Who will have access to the data?

Response:

The PI, study coordinator and statistician.

20.4 A. Who is responsible for receipt or transmission of the data?

Response:

The PI.

20.5 A. How will the data be transported?

Response:

Electronically.

B. Confidentiality of Study Specimens

Describe the local procedures for maintenance of confidentiality of ***study specimens****.*

☒ **N/A:** No specimens will be collected or analyzed in this research.
(Skip to Section 21.0)

20.6 B. Where and how will all specimens be stored? Include information about: physical controls, authorization of access, and labeling of specimens, as applicable.

Response:

20.7 B. How long will the specimens be stored?

Response:

20.8 B. Who will have access to the specimens?

Response:

20.9 B. Who is responsible for receipt or transmission of the specimens?

Response:

20.10 B. How will the specimens be transported?

Response:

21.0 Provisions to Monitor the Data to Ensure the Safety of Subjects*

☐ **N/A:** This study is not enrolling subjects, or is limited to records review procedures only. This section does not apply.

NOTE: Minimal risk studies may be required to monitor subject safety if the research procedures include procedures that present unique risks to subjects that require monitoring. Some examples include: exercising to exertion, or instruments that elicit suicidality or substance abuse behavior. In such cases, N/A is not an acceptable response.

21.1 Describe the plan to periodically evaluate the data collected regarding both harms and benefits to determine whether subjects remain safe.

Response:

Enrolled patients will be provided with the study teams' contact information as well as the UBMD Neurology call service phone number to be used on weekends and after business hours during the week in order to report any adverse events they may be experiencing during the study. If patients experience any adverse events at three study pills/day, they can decrease the dose back to two pills/day. Based on the lack of any side effects among the 11 long COVID patients taking

these dosages of lithium for one-six months, it is unlikely that there will be concerning side effects from lithium among the 50 patients in this study.

21.2 Describe what data are reviewed, including safety data, untoward events, and efficacy data.

Response:

Patient adverse events.

21.3 Describe any safety endpoints.

Response:

Total number of adverse events and individual adverse events in each treatment arm. Adverse events occurring in >10% of patients in a treatment arm will be noted.

21.4 Describe how the safety information will be collected (e.g., with case report forms, at study visits, by telephone calls with participants).

Response:

Adverse events will be collected by phone and inquired at Visit 2.

21.5 Describe the frequency of safety data collection.

Response:

The only formal inquiry of adverse events will be at Visit 2. However, patients can contact the study team by phone at any time during the study to report adverse events.

21.6 Describe who will review the safety data.

Response:

The PI will review AEs monthly. The safety monitor, Daniel Sirica, MD, will review AEs quarterly.

21.7 Describe the frequency or periodicity of review of cumulative safety data.

Response:

Monthly.

21.8 Describe the statistical tests for analyzing the safety data to determine whether harm is occurring.

Response:

None.

21.9 Describe any conditions that trigger an immediate suspension of the research.

Response:

- For non-serious subject adverse events:

Subjects will be directly asked by one of the study team members about any adverse events at Visit 2. All adverse events (AE's) will be recorded on the AE report form. Non-serious adverse events will be categorized as follows:

- **Mild** – Events require minimal or no treatment and do not interfere with the participant's daily activities.
- **Moderate** – Events result in a low level of inconvenience or concern with the therapeutic measures. Moderate events may cause some interference with functioning.
- **Severe** – Events interrupt a participant's usual daily activity and may require systemic drug therapy or other treatment. Severe events are usually potentially life-threatening or incapacitating. Of note, the term "severe" does not necessarily equate to "serious".

- For <u>serious</u> subject adverse events (SAE):

  An adverse event will be considered "serious" (SAE) if the adverse event resulted in subject death, a life-threatening adverse event, inpatient hospitalization or prolongation of existing hospitalization, or a persistent or significant incapacity or substantial disruption of the ability to conduct normal life functions. This is the definition of SAE according to the FDA's Guidance for Industry and Investigators: Safety Reporting Requirements for INDs and BA/BE Studies. When a study team member becomes aware of a possible subject SAE, the study team member will contact the PI by phone the same day.

  An SAE that is determined by the study PI to be, a) unexpected, and b) reasonably possible to have been caused by lithium will be considered a "Reportable Event". The PI will report such events to the University at Buffalo's IRB within 7 calendar days of the study team's first awareness of the event.

  An SAE that is unexpected by virtue of it not being listed in either the FDA approved product package insert, protocol or consent form with regard to occurrence or at an increased frequency, severity or duration than previously published will be considered a "Reportable Event". FDA Guidance from 2012 notes that an event should be reported if:

  -A single occurrence of an event that is uncommon and known to be strongly associated with drug exposure (e.g., angioedema, hepatic injury, Stevens-Johnson Syndrome)

  -One or more occurrences of an event that is not commonly associated with drug exposure, but is otherwise uncommon in the population exposed to the drug (e.g., tendon rupture)

  -An aggregate analysis of specific events observed in a clinical trial (such as known consequences of the underlying disease or condition under investigation or other events that commonly occur in the study population independent of drug therapy) that indicates those events occur more frequently in the drug treatment group than in a concurrent or historical control group.

- If similar "Reportable" SAEs occur in five subjects, the statistician will be unblinded to the treatment allocation for these subjects and alert the PI if all five are receiving lithium. If so, the study will be terminated and subjects instructed to stop lithium therapy.
- No interim data analysis will be performed.
- This study will be registered on clinicaltrials.gov prior to subject enrollment.

22.0 Withdrawal of Subjects*

☐ **N/A:** This study is not enrolling subjects. This section does not apply.

*22.1 Describe **anticipated** circumstances under which subjects may be withdrawn from the research without their consent.*

Response:

Subjects experiencing a "reportable" SAE (defined above) will be withdrawn.

22.2 Describe any procedures for orderly termination.

NOTE: Examples may include return of study drug, exit interview with clinician. Include whether additional follow up is recommended for safety reasons for physical or emotional health.

Response:

Subjects withdrawn from the double-blind phase of the study will be asked to make a final study visit during which all Visit 2 procedures will take place. Subjects withdrawn from the open-label phase will be asked to complete the questionnaires at home and mail them to the study team in the provided self-addressed, stamped envelope.

22.3 Describe procedures that will be followed when subjects withdraw from the research, including retention of already collected data, and partial withdrawal from procedures with continued data collection, as applicable.

Response:

All data provided by subjects withdrawn from the study will be retained and used in the final analysis.

23.0 Risks to Subjects*

23.1 List the reasonably foreseeable risks, discomforts, hazards, or inconveniences to the subjects related to their participation in the research. Consider physical, psychological, social, legal, and economic risks. Include a description of the probability, magnitude, duration, and reversibility of the risks.

NOTE: Breach of confidentiality is always a risk for identifiable subject data.

Response:

Side effects from lithium are typically related to the dosage taken. Because this study will be using low dosages of lithium (10-15mg/day), we anticipate a lower risk of side effects than when lithium is given in the higher dosages used for treating bipolar disorder (about 300mg/day). When used at higher dosages for treating bipolar disorder, lithium can cause the following side effects: hand tremors, increased urination, increased thirst, diarrhea, vomiting, drowsiness and lack of coordination. None of the 11 long COVID patients who have taken low-dose lithium (10-15mg/day) has reported any side effects to date.

Questionnaires: filling out the questionnaires or answering the study doctor or study staff's questions could lead patients to feel uncomfortable or upset.

23.2 Describe procedures performed to lessen the probability or magnitude of risks, including procedures being performed to monitor subjects for safety.

Response:

If patients get side effects at three pills/day, the dose can be decreased to two pills/day.

Patients will be instructed to tell the study doctor or study staff if they feel uncomfortable or upset while filling out a questionnaire or answering questions so that they can be counselled and their concerns addressed.

23.3 If applicable, indicate ***which procedures*** *may have risks to the subjects that are currently unforeseeable.*

Response:

Since the study drug is investigational for treating long COVID, there may be other risks that are unknown. Patients will be informed in a timely manner if new information about the study becomes available that may be relevant to continued participation in the trial. All drugs have a potential risk of an allergic reaction, which if not treated promptly, could become life-threatening. Some things that happen during an allergic reaction that could be a sign or symptom of a life-threatening allergic reaction (anaphylaxis) are:

- a rash
- a fast pulse
- sweating
- a feeling of dread
- swelling around the eyes and mouth
- swelling of the throat
- wheezing
- having a hard time breathing
- a sudden drop in blood pressure (making you feel dizzy or lightheaded)
- inability to breathe without assistance

Patients will be informed to get medical help and contact the study doctor or study staff if they have any of these or any other side effects during the study.

23.4 If applicable, indicate which research procedures may have risks to an embryo or fetus should the subject be or become pregnant.

Response:

Lithium therapy is associated with higher rates of birth defects. Therefore, women of child bearing potential must have a negative pregnancy test to be eligible for this study. In order to reduce the risk of pregnancy during the study, women of child bearing potential must use an effective method of birth control while participating in this study.

Acceptable methods of birth control include oral contraceptive or long-term injectable or implantable hormonal contraceptive, double-barrier methods such as condom plus diaphragm, condom plus spermicide foam, condom plus sponge, or intra-uterine devices or abstinence. Oral, implantable, or injectable contraceptives are only considered effective if used properly and started at least 30 days prior to the screening visit. Some drugs (e.g., antibiotics) may interact with hormonal contraceptives, making them not work properly.

23.5 If applicable, describe risks to others who are not subjects.

Response:

None.

24.0 Potential Benefits to Subjects*

24.1 Describe the potential benefits that individual subjects may experience by taking part in the research. Include the probability, magnitude, and duration of the potential benefits. Indicate if there is no direct benefit.

*NOTE: Compensation **cannot** be stated as a benefit.*

Response:

Patients may notice improvements in their long COVID symptoms.

25.0 Compensation for Research-Related Injury

☐ **N/A:** The research procedures for this study do not present risk of research related injury (e.g. survey studies, records review studies). This section does not apply.

25.1 ***If the research procedures carry a risk of research related injury,*** *describe the available compensation to subjects in the event that such injury should occur.*

Response:

Subjects will receive medical treatment if injured or become ill as a result of this study.

The University at Buffalo makes no commitment to provide free medical care or payment for any unfavorable outcomes that result from your participation in this research. Medical services will be billed at the usual charge and will be the subject's responsibility or that of a third-party payer but subjects will not be precluded from seeking to collect compensation for injury related to malpractice, fault, or blame on the part of those involved in the research including the University at Buffalo.

By accepting medical care or accepting payment for medical expenses, subjects are not waiving any legal rights.

25.2 Provide a copy of contract language, if any, relevant to compensation for research related injury.

*NOTE: If the contract is not yet approved at the time of this submission, submit the current version here. If the contract is later approved with **different language regarding research related injury**, you must modify your response here and submit an amendment to the IRB for review and approval.*

Response:

N/A

26.0 Economic Burden to Subjects

26.1 Describe any costs that subjects may be responsible for because of participation in the research.

NOTE: Some examples include transportation or parking.

Response:

Transportation costs. Parking is free.

☐ **N/A:** This study is not enrolling subjects, or is limited to records review procedures only. This section does not apply.

27.0 Compensation for Participation

27.1 Describe the amount and timing of any compensation to subjects, including monetary, course credit, or gift card compensation.

Response:

☐ **N/A:** This study is not enrolling subjects, or is limited to records review procedures only. This section does not apply.

☒ **N/A:** There is no compensation for participation. This section does not apply.

28.0 Consent Process

28.1 Indicate whether you will be obtaining consent.

NOTE: This does not refer to consent documentation, but rather whether you will be obtaining permission from subjects to participate in a research study. Consent documentation is addressed in Section 29.0.

☒ ***Yes*** *(If yes, Provide responses to each question in this Section)*
☐ ***No*** *(If no, Skip to Section 29.0)*

28.2 Describe where the consent process will take place. Include steps to maximize subjects' privacy.

Response:

The consent process will occur in a closed-door, examination clinic room at UBMD Neurology.

28.3 Describe how you will ensure that subjects are provided with a sufficient period of time to consider taking part in the research study.

NOTE: It is always a requirement that a prospective subject is given sufficient time to have their questions answered and consider their participation. See "SOP: Informed Consent Process for Research (HRP-090)" Sections 5.5 and 5.6.

Response:

Potential subjects will be provided with the consent document at least 48 hours prior to the screening visit to ensure adequate time to read and process the entire document.

28.4 Describe any process to ensure ongoing consent, defined as a subject's willingness to continue participation for the duration of the research study.

Response:

Subjects will be asked if they wish to continue in the study at each study visit and phone call.

28.5 Indicate whether you will be following "SOP: Informed Consent Process for Research (HRP-090)." Pay particular attention to Sections 5.4-5.9. If not, or if there are any exceptions or additional details to what is covered in the SOP, describe:

- *The role of the individuals listed in the application who are involved in the consent process*
- *The time that will be devoted to the consent discussion*
- *Steps that will be taken to minimize the possibility of coercion or undue influence*
- *Steps that will be taken to ensure the subjects' understanding*

Response:

☒ We have reviewed and will be following "SOP: Informed Consent Process for Research (HRP-090)."

Non-English Speaking Subjects

☒ **N/A:** This study will not enroll Non-English speaking subjects. *(Skip to Section 28.8)*

28.6 Indicate which language(s) other than English are likely to be spoken/understood by your prospective study population or their legally authorized representatives.

NOTE: The response to this Section should correspond with your response to Section 8.4 of this protocol.

Response:

28.7 If subjects who do not speak English will be enrolled, describe the process to ensure that the oral and written information provided to those subjects

will be in that language, how you will ensure that subjects are provided with a sufficient period of time to consider taking part in the research study, and any process to ensure ongoing consent. Indicate the language that will be used by those obtaining consent.

NOTE: Guidance is provided on "SOP: Informed Consent Process for Research (HRP-090)."

Response:

Cognitively Impaired Adults

☐ **N/A**: This study will not enroll cognitively impaired adults.
(Skip to Section 28.9)

28.8 Describe the process to determine whether an individual is capable of consent.

Response:

After subjects have had adequate time to review the consent and ask questions about the study, they will be asked to give a brief overview of the study procedures. Only subjects that can accurately describe key aspects of the study (i.e. that they will receive either lithium or a placebo for the first three weeks in a double-blind fashion and then return to the clinic for a final visit when they will receive lithium to use for two additional weeks; that they can withdraw from the study at any time) will be enrolled.

Adults Unable to Consent

☒ **N/A**: This study will not enroll adults unable to consent.
(*Skip to Section 28.13)*

When a person is not capable of consent due to cognitive impairment, a legally authorized representative should be used to provide consent (Sections 28.9 and 28.10) ***and, where possible, assent of the individual should also be solicited*** *(Sections 28.11 and 28.12).*

28.9 Describe how you will identify a Legally Authorized Representative (LAR). Indicate that you have reviewed the "SOP: Legally Authorized Representatives, Children, and Guardians (HRP-013)" for research in New York State.

NOTE: Examples of acceptable response includes: verifying the electronic medical record to determine if an LAR is recorded.

Response:

☐ We have reviewed and will be following "SOP: Legally Authorized Representatives, Children, and Guardians (HRP-013)."

28.10 ***For research conducted outside of New York State****, provide information that describes which individuals are authorized under applicable law to consent on behalf of a prospective subject to their participation in the research. One method of obtaining this information is to have a legal counsel or authority review your protocol along with the definition of "legally authorized representative" in "SOP: Legally Authorized Representatives, Children, and Guardians (HRP-013)."*

Response:

28.11 Describe the process for ***assent of the adults****:*

- *Indicate whether assent will be obtained from all, some, or none of the subjects.* ***If some, indicate which adults will be required to assent and which will not.***

Response:

- ***If assent will not be obtained from some or all subjects, provide an explanation of why not.***

Response:

28.12 Describe whether ***assent of the adult*** *subjects will be documented and the process to document assent.*

NOTE: The IRB allows the person obtaining assent to document assent on the consent document using the "Template Consent Document (HRP-502)" Signature Block for Assent of Adults who are Legally Unable to Consent.

Response:

Subjects who are not yet Adults (Infants, Children, and Teenagers)

☒ **N/A**: This study will not enroll subjects who are not yet adults.
(Skip to Section 29.0)

28.13 Describe the criteria that will be used to determine ***whether a prospective subject has not attained the legal age for consent to treatments or procedures involved in the research*** *under the applicable law of the jurisdiction in which the research will be conducted* ***(e.g., individuals under the age of 18 years)****. For research conducted in NYS, review "SOP: Legally Authorized Representatives, Children, and Guardians (HRP-013)"*

to be aware of which individuals in the state meet the definition of "children."

NOTE: Examples of acceptable responses include: verification via electronic medical record, driver's license or state-issued ID, screening questionnaire.

Response:

28.14 ***For research conducted outside of New York State****, provide information that describes which persons have not attained the legal age for consent to treatments or procedures involved the research, under the applicable law of the jurisdiction in which research will be conducted. One method of obtaining this information is to have a legal counsel or authority review your protocol along the definition of "children" in "SOP: Legally Authorized Representatives, Children, and Guardians (HRP-013)."*

Response:

28.15 Describe whether parental permission will be obtained from:

Response:

☐ One parent even if the other parent is alive, known, competent, reasonably available, and shares legal responsibility for the care and custody of the child.

☐ Both parents unless one parent is deceased, unknown, incompetent, or not reasonably available, or when only one parent has legal responsibility for the care and custody of the child.

☐ Parent permission will not be obtained. A waiver of parent permission is being requested.

NOTE: The requirement for parent permission is a protocol-specific determination made by the IRB based on the risk level of the research. For guidance, review the "CHECKLIST: Children (HRP-416)."

28.16 Describe whether permission will be obtained from individuals ***other than parents****, and if so, who will be allowed to provide permission. Describe your procedure for determining an individual's authority to consent to the child's general medical care.*

Response:

28.17 Indicate whether assent will be obtained from all, some, or none of the ***children****. If assent will be obtained from some children, indicate which children will be required to assent.*

Response:

28.18 When assent of children is obtained, describe how it will be documented.

Response:

29.0 Waiver or Alteration of Consent Process

Consent will not be obtained, required information will not be disclosed, or the research involves deception.

☒ **N/A:** A waiver or alteration of consent is not being requested.

29.1 If the research involves a waiver or alteration of the consent process, please review the "CHECKLIST: Waiver or Alteration of Consent Process (HRP-410)" to ensure that you have provided sufficient information for the IRB to make the determination that a waiver or alteration can be granted.

NOTE: For records review studies, the first set of criteria on the "CHECKLIST: Waiver or Alteration of Consent Process (HRP-410)" applies.

Response:

29.2 If the research involves a waiver of the consent process for planned emergency research, please review the "CHECKLIST: Waiver of Consent for Emergency Research (HRP-419)" to ensure you have provided sufficient information for the IRB to make these determinations. Provide any additional information necessary here:

Response:

30.0 Process to Document Consent

☐ **N/A:** A Waiver of Consent is being requested.
(Skip to Section 31.0)

30.1 Indicate whether you will be following "SOP: Written Documentation of Consent (HRP-091)." If not or if there are any exceptions, describe whether and how consent of the subject will be obtained including whether or not it will be documented in writing.

NOTE: If your research presents no more than minimal risk of harm to subjects and involves no procedures for which written documentation of consent is normally required outside of the research context, the IRB will generally waive the requirement to obtain written documentation of consent. This is sometimes referred to as 'verbal consent.' Review "CHECKLIST: Waiver of Written Documentation of Consent (HRP-411)" to ensure that you have provided sufficient information.

If you will document consent in writing, attach a consent document with your submission. You may use "TEMPLATE CONSENT DOCUMENT (HRP-

502)". If you will obtain consent, but not document consent in writing, attach the script of the information to be provided orally or in writing (i.e. consent script or Information Sheet).

Response:

☒ We will be following "SOP: Written Documentation of Consent" (HRP-091).

31.0 Multi-Site Research (Multisite/Multicenter Only)*

☒ **N/A:** This study is not an investigator-initiated multi-site study. This section does not apply.

31.1 Indicate the total number of subjects that will be enrolled or records that will be reviewed across all sites.

Response:

*31.2 If this is a multi-site study **where you are the lead investigator**, describe the processes to ensure communication among sites, such as the following.*

- *All sites have the most current version of the IRB documents, including the protocol, consent document, and HIPAA authorization.*
- *All required approvals have been obtained at each site (including approval by the site's IRB of record).*
- *All modifications have been communicated to sites, and approved (including approval by the site's IRB of record) before the modification is implemented.*
- *All engaged participating sites will safeguard data as required by local information security policies.*
- *All local site investigators conduct the study appropriately in accordance with applicable federal regulations and local laws.*
- *All non-compliance with the study protocol or applicable requirements will be reported in accordance with local policy.*

Response:

31.3 Describe the method for communicating to engaged participating sites.

- *Problems (inclusive of reportable events)*
- *Interim results*
- *Study closure*

Response:

*31.4 If this is a multicenter study **where you are a participating site/investigator**, describe the local procedures for maintenance of confidentiality.*

- *Where and how data or specimens will be stored locally?*
- *How long the data or specimens will be stored locally?*
- *Who will have access to the data or specimens locally?*
- *Who is responsible for receipt or transmission of the data or specimens locally?*
- *How data and specimens will be transported locally?*

Response:

31.5 If this is a multicenter study and subjects will be recruited by methods not under the control of the local site (e.g., call centers, national advertisements) describe those methods. Local recruitment methods are described elsewhere in the protocol.

- *Describe when, where, and how potential subjects will be recruited.*
- *Describe the methods that will be used to identify potential subjects.*
- *Describe materials that will be used to recruit subjects. (Attach copies of these documents with the application. For advertisements, attach the final copy of printed advertisements. When advertisements are taped for broadcast, attach the final audio/video tape. You may submit the wording of the advertisement prior to taping to preclude re-taping because of inappropriate wording, provided the IRB reviews the final audio/video tape.)*

Response:

32.0 Banking Data or Specimens for Future Use*

☐ **N/A:**

*32.1 If data or specimens will be banked (stored) for **future use, that is, use or research outside of the scope of the present protocol**, describe where the data/specimens will be stored, how long they will be stored, how the data/specimens will be accessed, and who will have access to the data/specimens.*

NOTE: Your response here must be consistent with your response at the "What happens if I say yes, I want to be in this research?" Section of the Template Consent Document (HRP-502).

NOTE: If the UBIRB has approved this study to bank data and/or specimens for potential future use outside the scope of this research study, any future use or

disclosure of the data that is not described within the approved study must be submitted for review to the UBIRB.

Response: Patient deidentified data will be stored in a password-protected excel file indefinitely. The study PI will have access to the data.

32.2 List the data to be stored or associated with each specimen.

Response: Questionnaire data and deidentified patient demographic data.

32.3 Describe the procedures to release banked data or specimens for future uses, including: the process to request a release, approvals required for release, who can obtain data or specimens, and the data to be provided with specimens.

Response: The PI will grant data access upon reasonable written request from a reputable research team.
