# Supplementary material for: Lithium Aspartate for Long COVID Fatigue and Cognitive Dysfunction: A Randomized Clinical Trial
Source: JAMA Netw Open. 2024 Oct 2;7(10):e2436874. doi: 10.1001/jamanetworkopen.2024.36874 (PMC11447566; doi:10.1001/jamanetworkopen.2024.36874)
Supplement: Supplement 2. — Dose-Finding Study Protocol [file jamanetwopen-e2436874-s002.pdf]

University at Buffalo
The State University of New York

University at Buffalo Institutional Review Board (UBIRB)
Office of Research Compliance | Clinical and Translational Research Center Room 5018
875 Ellicott St. | Buffalo, NY 14203
UB Federalwide Assurance ID#: FWA00008824

Complete Research Protocol (HRP-503)

Table of Contents

Template Instructions 2
1.0 Study Summary 4
2.0 Objectives* 4
3.0 Scientific Endpoints* 5
4.0 Background* 5
5.0 Study Design* 10
6.0 Study Intervention/Investigational Agent 10
7.0 Local Number of Subjects 11
8.0 Inclusion and Exclusion Criteria* 11
9.0 Vulnerable Populations* 13
10.0 Eligibility Screening* 14
11.0 Recruitment Methods 14
12.0 Procedures Involved* 16
13.0 Study Timelines* 18
14.0 Setting 18
15.0 Community-Based Participatory Research 19
16.0 Resources and Qualifications 19
17.0 Other Approvals 20
18.0 Provisions to Protect the Privacy Interests of Subjects 21
19.0 Data Management and Analysis* 21
20.0 Confidentiality* 22
A. Confidentiality of Study Data 22
B. Confidentiality of Study Specimens 23
21.0 Provisions to Monitor the Data to Ensure the Safety of Subjects* 24
22.0 Withdrawal of Subjects* 26
23.0 Risks to Subjects* 27
24.0 Potential Benefits to Subjects* 28
25.0 Compensation for Research-Related Injury 28
26.0 Economic Burden to Subjects 29
27.0 Compensation for Participation 29
28.0 Consent Process 30
29.0 Waiver or Alteration of Consent Process 34
30.0 Process to Document Consent 35
31.0 Multi-Site Research (Multisite/Multicenter Only)* 35
32.0 Banking Data or Specimens for Future Use* 37

Template Instructions

Sections that do not apply:

- *In several sections, the addition of checkboxes for* **Not Applicable** *have been added to the template as responses.*
    - *If an N/A checkbox is present, select the appropriate justification from the list.*
    - *If an N/A checkbox is not present, or if none of the existing checkboxes apply to your study, you must write in your own justification.*
- *In addition:*
    - *For research where the only study procedures are records/chart review: Sections 6, 21, 22, 24, 25, 26 and 27 do not apply.*
    - *For exempt research: Section 6 may not apply. Section 6.1 will still apply if there is a study intervention.*

Studies with multiple participant groups:

- *If this study involves multiple participant groups (e.g. parents and children), provide information in applicable sections for each participant group. Clearly label responses when they differ. For example:*

  Response Example

  <u>Intervention Group:</u> Lithium

Formatting:

- *Do not remove template instructions or section headings when they do not apply to your study.*

If you are pasting information from other documents using the "Merge Formatting" Paste option will maintain the formatting of the response boxes.

Amendments:

- *When making modifications or revisions to this and other documents, use the* ***Track Changes*** *function in Microsoft Word.*
- *Update the version date or number* ***on Page 3.***

PROTOCOL TITLE:

Include the full protocol title.

Response: Effect of low-dose lithium therapy on long COVID symptoms: an open-label, dose-finding trial.

PRINCIPAL INVESTIGATOR:

Thomas Guttuso, Jr., MD; Department of Neurology; 716-932-6080; tguttuso@buffalo.edu

VERSION NUMBER/DATE:

Include the version number and date of this protocol.

Response: Version 1, 10/6/23

REVISION HISTORY

Revision #	Version Date	Summary of Changes	Consent Change?

FUNDING:

Indicate any funding for this proposal. This should match the Funding Sources page in Click IRB.

Response: Internal UB Department of Neurology funds.

GRANT APPLICABILITY:

Indicate whether this protocol is funded by a grant (e.g. NIH, foundation grant). For a grant with multiple aims, indicate which aims are covered by this research proposal.

NOTE: This question does not apply to studies funded by a sponsor contract.

Include a copy of the grant proposal with your submission.

Response: This study will not be funded by a grant.

RESEARCH REPOSITORY:

Indicate where the research files will be kept, including when the study has been closed. The repository should include, at minimum, copies of IRB correspondence (approval, determination letters) as well as signed consent documents. This documentation should be maintained for 3 years after the study has been closed.

Location: UBMD Neurology

Address: 5851 Main Street, Williamsville, NY 14221

Department: Neurology

1.0 Study Summary

**Study Title**	Effect of low-dose lithium therapy on long COVID symptoms: an open-label, dose-finding study
**Study Design**	Open-label
**Primary Objective**	To determine if higher lithium dosages than used in the previous double-blind or open-label study phases are associated with more benefit to long-COVID symptoms.
**Secondary Objective(s)**	To determine the lithium serum levels corresponding to maximum symptomatic benefit.
**Research Intervention(s)/ Investigational Agent(s)**	Lithium
**IND/IDE #**	IND exempt per FDA Guidance
**Study Population**	Long COVID patients
**Sample Size**	50
**Study Duration for individual participants**	6-11 weeks.
**Study Specific Abbreviations/ Definitions**	

2.0 Objectives*

2.1 Describe the purpose, specific aims, or objectives of this research.

Response: To determine if higher lithium dosages than used in the previous double-blind or open-label study phases are associated with more benefit to long-COVID symptoms. To determine the lithium serum levels corresponding to maximum symptomatic benefit.

2.2 State the hypotheses to be tested, if applicable.

NOTE: A hypothesis is a specific, testable prediction about what you expect to happen in your study that corresponds with your above listed objectives.

Response: Open-label lithium therapy at 20-45mg/day will be associated with greater improvements in long COVID fatigue and/or brain fog than what each individual patient previously reported when receiving open-label lithium therapy at 15mg/day.

3.0 Scientific Endpoints*

3.1 Describe the scientific endpoint(s), the main result or occurrence under study.

NOTE: Scientific endpoints are outcomes defined before the study begins to determine whether the objectives of the study have been met and to draw conclusions from the data. Include primary and secondary endpoints. Some example endpoints are: reduction of symptoms, improvement in quality of life, or survival. Your response should ***not*** *be a date.*

Response:

Primary Endpoint:

Change from Visit 1 to Visit 2 in combined Fatigue Severity Scale (FSS) and Brain Fog Severity Scale (BFSS) scores.

Secondary Endpoints:

For the proposed open-label, lithium dose-finding extension study, individual patient outcomes will be compared to those reported from previous long COVID study when each patient received open-label lithium 15mg/day. Outcomes include change from new visit 1 to visit 2 in combined Fatigue Severity Scale (FSS) and Brain Fog Severity Scale (BFSS) scores; Well-Being Scale scores; Short Form-12 Health Survey scores (with 1-week modification); FSS scores in those with FSS score ≥28 at baseline; BFSS scores in those with BFSS score ≥28 at baseline; Modified Fatigue Impact Scale, Perceived Deficits Questionnaire 5-Item Version, Beck Depression Inventory-II; Generalized Anxiety Disorder Scale scores in those with score ≥1 at baseline; Headache and Body Pain Bother Scale scores in those with score ≥2 at baseline; Insomnia Severity Index scores in those with score ≥10 at baseline; PGIC and Desire to Continue Therapy Scale scores at visit 2.

4.0 Background*

4.1 Provide the scientific or scholarly background, rationale, and significance of the research based on the existing literature and how it will contribute to existing knowledge. Describe any gaps in current knowledge. Include relevant preliminary findings or prior research by the investigator.

Response:

Long COVID, also known as long-haul COVID or post-acute sequelae of SARS CoV-2 infection (PASC), is defined by the Centers for Disease Control and Prevention (CDC) as having persistent symptoms for >4 weeks after recovering from the infectious phase of COVID-19. The most common long COVID symptoms are fatigue, concentration/cognitive impairment (also called “brain fog”) and shortness of breath with exertion. In addition, other long COVID symptoms include dizziness when standing,

chronic headaches, diffuse muscle and/or joint pains, abdominal pain, decreased sense of smell or taste, coordination problems, insomnia, anxiety and depression.[1] The long COVID symptoms stemming from the brain- such as fatigue, brain fog, headaches, etc.- have been coined "neuro long COVID" symptoms. It is estimated that about 20-30% of people infected with COVID develop long COVID with varying degrees of severity.[2] About 30 million people in the US are estimated to have long COVID and over one million are currently disabled from it.

Although the cause of neuro long COVID is currently unknown, evidence supports increased neuroinflammation as a contributor to the pathophysiology perhaps being triggered by COVID viral proteins persisting in brain cells.[3] A recent study showed significantly increased inflammatory markers of C-reactive protein (CRP) and serum amyloid A in the cerebrospinal fluid (CSF) of long COVID patients with brain fog compared to post-COVID patients without any long COVID.[4] In addition, there were trends towards increased CSF levels of several other inflammatory markers in neuro long COVID patients. There is also evidence that COVID-19-induced inflammation may reactivate the Epstein-Barr Virus (EBV) in long COVID patients and contribute to the symptom similarities between patients with long COVID and those with EBV reactivation.[5]

However, it does not appear that increased brain inflammation is reflected in the systemic circulation of long COVID patients. One study did report significantly increased blood acute phase reactants; such as erythrocyte sedimentation rate (ESR), c-reactive protein (CRP), D-dimer and ferritin; among 120 patients who had tested positive for COVID-19 about 4 months previously compared to 120 matched control patients; however, this study did not assess patients for long COVID symptoms.[6] A more recent study enrolled 189 patients between July 2020 and July 2021 who had tested positive for COVID-19 about 5 months previously including 104 patients with long COVID.[7] This study found no significant differences between either the whole COVID-19 cohort or the long COVID subgroup and 120 control patients who never had COVID for blood CRP, D-dimer or plasma neurofilament light or detection of antinuclear antibody (ANA), anticardiolipin antibody or rheumatoid factor antibody. Another study reported an increase in several proinflammatory cytokines in the serum of post-COVID patients compared to controls but with no differences observed between long COVID and post-COVID patients without long COVID symptoms.[8] Thus, the current body of evidence has yet to identify any serum biomarkers for long COVID.

Because increased brain inflammation may be involved in the pathophysiology of neuro long COVID and microglia are the primary immune cell in the brain mediating neuroinflammation, therapies known to cross the blood-brain barrier (BBB) and suppress microglial activation could potentially be effective for treating patients with neuro long COVID.

Lithium has been shown to effectively suppress microglial activation and reduce serum inflammatory cytokines including interleukin-6 (IL-6) and tumor necrosis factor-alpha (TNF-a).[9-12] Lithium is well known to be centrally active as a highly effective treatment for bipolar disorder and readily crosses the BBB and cell membranes via passage through sodium channels.[13,14] Although the elemental lithium dosage required to treat bipolar disorder is about 300mg/day, there is clinical evidence that dosages <1mg/day may prevent and slow Alzheimer's disease (AD) and Parkinson's disease (PD).[15-17] Chronic brain inflammation has been implicated in the pathophysiology of both AD and PD.[18]

The term "elemental lithium" refers to the weight of lithium alone not including the weight of the salt carrier bound to lithium such as the carbonate moiety in prescription lithium carbonate or the aspartate or orotate moieties in over-the-counter (OTC) lithium

dietary supplement products. The OTC lithium dietary supplement products available are lithium aspartate and lithium orotate, which typically provide 5mg of elemental lithium/capsule. Because orotate has been shown to increase the incidences of several cancers in rodent cancer models,[19,20] we chose to study the effects of lithium aspartate on blood-based and MRI biomarkers in a recent pilot PD clinical trial.

Results showed that in PD patients receiving about 150mg/day of elemental lithium as lithium carbonate or 45mg/day or 15mg/day of elemental lithium as lithium aspartate for six months, mean trough serum lithium levels were 1282, 828 and 195µg/L, respectively, compared to 0.73µg/L in PD patients not receiving lithium.[21] These data confirm that OTC lithium aspartate contains bioavailable elemental lithium. In addition, the results from the lithium/PD pilot study showed lithium aspartate 45mg/day to be associated with the largest and most consistent reductions in brain inflammation reflected by reductions in brain free water levels assessed by diffusion-weighted MRI. Lithium aspartate 15mg/day was not associated with reductions in brain free water levels. Thus, lithium dosages higher than 15mg/day may be required to provide symptomatic benefits for brain conditions stemming from chronic inflammation such as neuro long COVID.

Preliminary data:

Because neuroinflammation may be involved in long COVID pathophysiology and low-dose lithium may have neuroprotective effects in neurodegenerative diseases that are known to have increased neuroinflammation, lithium aspartate 5mg/day was suggested by Dr. Guttuso in March 2022 for use in a 17-year-old male with long COVID. This patient had severe fatigue, brain fog and daily headaches for 10 weeks after testing positive for COVID-19 and had failed therapy with prednisone and baclofen for these symptoms. These symptoms were disabling as the patient was unable to attend high school or lacrosse practices and spent most of the day lying in bed, according to his parents. Within two days of starting lithium aspartate 5mg/day, the patient reported about 90% improvements in all three of his neuro long COVID symptoms and was able to return to classes. Three weeks later he developed another viral infection and tested negative for COVID but had recurrences of his neuro long COVID symptoms. He increased lithium aspartate to 10mg/day and again reported about 90% improvements in these symptoms within 3-5 days. Encouraged by this anecdotal case, lithium aspartate was recommended to eight additional patients with neuro long COVID symptoms for at least six weeks after testing positive for COVID-19. Most but not all of these patients have reported some degree of benefit to their long COVID symptoms after starting low-dose lithium therapy. Anecdotally, it appeared that the symptoms of fatigue and brain fog improved to the greatest extent. No side effects have been reported by any patient that could potentially be from lithium.

These preliminary reports justified conducting a randomized controlled trial (RCT) to formally assess the effectiveness of lithium aspartate 15mg/day on the neuro long COVID symptoms of fatigue and brain fog. From November 2022 to July 2023, 52 patients with moderate-severe long COVID fatigue and/or brain fog were enrolled by Dr. Guttuso into a RCT at UB. Two patients were lost-to-follow-up and 50 completed the 3-week double-blind study phase. All 50 patients then entered a 2-week open-label lithium aspartate 15mg/day treatment phase after which study questionnaires were completed at home and mailed to the PI. Forty-five patients completed the open-label questionnaires.

There were no patient withdrawals. Six patients reported side effects during the double-blind study phase. Four patients receiving placebo reported side effects of acne, thyroid goiter, increased seasonal allergies and increased headaches, respectively. The

patient with the goiter reported that this was known by her PMD prior to her enrolling in this study; however, her PMD did not inform her of this until after she was enrolled. Two patients receiving lithium reported side effects of leg cellulitis (which was a recurrent problem for many years previously) and a foot fracture from an accident on a stair, respectively. No patients reported side effects during open-label treatment with lithium aspartate 15mg/day.

Results showed no significant intergroup differences for any of the primary or secondary outcomes. There were slightly more improvements in fatigue and brain fog scores in the lithium group compared to the placebo group but all with *p*-values > 0.30 (*unpublished data*). The responder analysis showed that patients reporting their long COVID symptoms to be "Much Improved" or "Very Much Improved" on the PGIC had 95% confidence interval reductions in FSS and BFSS scores of 17.4-32.3 and 14.4-28.6, respectively.

Interestingly, one patient who reported no benefit in FSS or BFSS scores while receiving placebo for 3 weeks or open-label lithium aspartate 15mg/day for 2 weeks during the study subsequently reported marked benefits in both symptoms within 7 days after increasing the lithium aspartate dosage to 30mg/day, which he purchased OTC after completing the study. This patient has been taking this dosage for 8 weeks, to date, and continues to report marked and satisfactory benefits to fatigue and brain fog without side effects.

This patient's anecdotal experience suggests that higher lithium dosages may offer meaningful improvements to neuro long COVID symptoms that were not apparent at 15mg/day. If a therapy is required to reduce brain inflammation in order to significantly reduce neuro long COVID fatigue and brain fog, the MRI free water results from the lithium/PD trial offer mechanistic support that dosages > 15mg/day may be required. The present lithium dose-finding study seeks to gain preliminary data using lithium aspartate dosages of 20-45mg/day among the neuro long COVID patients previously enrolled in the UB long COVID trial for whom results using open-label lithium aspartate 15/day are already available for comparison. Serum lithium levels will also be assessed to help determine an approximate therapeutic serum level for neuro long COVID symptoms. These patients will be contacted by email or mail and provided with the updated consent form to assess their interest in this lithium dose-finding extension phase. Those who express interest will be scheduled for a study visit. Results from this dose-finding study could provide preliminary data to support another neuro long COVID randomized controlled trial using lithium dosages >15mg/day.

4.2 Include complete citations or references.

Response:

1. Taquet M, Dercon Q, Luciano S, Geddes JR, Husain M, Harrison PJ. Incidence, co-occurrence, and evolution of long-COVID features: A 6-month retrospective cohort study of 273,618 survivors of COVID-19. PLoS Med 2021;18:e1003773.
2. Nehme M, Braillard O, Alcoba G, et al. COVID-19 Symptoms: Longitudinal Evolution and Persistence in Outpatient Settings. Ann Intern Med 2021;174:723-5.
3. Peluso MJ, Deeks SG, Mustapic M, et al. SARS-CoV-2 and Mitochondrial Proteins in Neural-Derived Exosomes of COVID-19. Ann Neurol 2022;91:772-81.
4. Medpagetoday. Post-COVID Brain Fog Tied to CSF Markers- Overstimulated immune system may be impetus for long COVID cognitive symptoms. Available at:

https://www.medpagetoday.com/meetingcoverage/aanvideopearls/98559 Accessed 20 July 2022.
5. Gold JE, Okyay RA, Licht WE, Hurley DJ. Investigation of Long COVID Prevalence and Its Relationship to Epstein-Barr Virus Reactivation. Pathogens 2021;10.
6. Gameil MA, Marzouk RE, Elsebaie AH, Rozaik SE. Long-term clinical and biochemical residue after COVID-19 recovery. Egypt Liver J 2021;11:74.
7. Sneller MC, Liang CJ, Marques AR, et al. A Longitudinal Study of COVID-19 Sequelae and Immunity: Baseline Findings. Ann Intern Med 2022.
8. Phetsouphanh C, Darley DR, Wilson DB, et al. Immunological dysfunction persists for 8 months following initial mild-to-moderate SARS-CoV-2 infection. Nat Immunol 2022;23:210-6.
9. Dong H, Zhang X, Dai X, et al. Lithium ameliorates lipopolysaccharide-induced microglial activation via inhibition of toll-like receptor 4 expression by activating the PI3K/Akt/FoxO1 pathway. Journal of neuroinflammation 2014;11:140.
10. Yu F, Wang Z, Tchantchou F, Chiu CT, Zhang Y, Chuang DM. Lithium ameliorates neurodegeneration, suppresses neuroinflammation, and improves behavioral performance in a mouse model of traumatic brain injury. J Neurotrauma 2012;29:362-74.
11. Valvassori SS, Tonin PT, Varela RB, et al. Lithium modulates the production of peripheral and cerebral cytokines in an animal model of mania induced by dextroamphetamine. Bipolar Disord 2015;17:507-17.
12. Spuch C, Lopez-Garcia M, Rivera-Baltanas T, et al. Efficacy and Safety of Lithium Treatment in SARS-CoV-2 Infected Patients. Front Pharmacol 2022;13:850583.
13. Janka Z, Jones DG. Lithium entry into neural cells via sodium channels: a morphometric approach. Neuroscience 1982;7:2849-57.
14. Lahteenvuo M, Tanskanen A, Taipale H, et al. Real-world Effectiveness of Pharmacologic Treatments for the Prevention of Rehospitalization in a Finnish Nationwide Cohort of Patients With Bipolar Disorder. JAMA Psychiatry 2018;75:347-55.
15. Guttuso T, Jr. High lithium levels in tobacco may account for reduced incidences of both Parkinson’s disease and melanoma in smokers through enhanced β-catenin mediated activity. Med Hypothesis 2019;131.
16. Kessing LV, Gerds TA, Knudsen NN, et al. Association of Lithium in Drinking Water With the Incidence of Dementia. JAMA Psychiatry 2017;74:1005-10.
17. Nunes MA, Viel TA, Buck HS. Microdose lithium treatment stabilized cognitive impairment in patients with Alzheimer's disease. Curr Alzheimer Res 2013;10:104-7.
18. Guzman-Martinez L, Maccioni RB, Andrade V, Navarrete LP, Pastor MG, Ramos-Escobar N. Neuroinflammation as a Common Feature of Neurodegenerative Disorders. Front Pharmacol 2019;10:1008.
19. Laconi E, Denda A, Rao PM, Rajalakshmi S, Pani P, Sarma DS. Studies on liver tumor promotion in the rat by orotic acid: dose and minimum exposure time required for dietary orotic acid to promote hepatocarcinogenesis. Carcinogenesis 1993;14:1771-5.

20. Laconi E, Vasudevan S, Rao PM, Rajalakshmi S, Pani P, Sarma DS. The effect of long-term feeding of orotic acid on the incidence of foci of enzyme-altered hepatocytes and hepatic nodules in Fischer 344 rats. Carcinogenesis 1993;14:1901-5.
21. Guttuso T, Jr., Shepherd R, Frick L, et al. Lithium's effects on therapeutic targets and MRI biomarkers in Parkinson's disease: A pilot clinical trial. IBRO Neurosci Rep 2023;14:429-34.

5.0 Study Design*

5.1 Describe and explain the study design (e.g. case-control, cross-sectional, ethnographic, experimental, interventional, longitudinal, observational).

Response:

Open-label, lithium dose-finding extension study.

6.0 Study Intervention/Investigational Agent

6.1 Describe the study intervention and/or investigational agent (e.g., drug, device) that is being evaluated.

Response:

Lithium aspartate

6.2 Drug/Device Handling: If the research involves drugs or device, describe your plans to store, handle, and administer those drugs or devices so that they will be used only on subjects and be used only by authorized investigators.

- *If the control of the drugs or devices used in this protocol will be accomplished by following an established, approved organizational SOP (e.g., Research Pharmacy SOP for the Control of Investigational Drugs, etc.), please reference that SOP in this section.*

Response:

Lithitate, NSF-certified lithium aspartate 5mg capsules, manufactured by e3 Pharmaceuticals, will be used for this dose-finding extension study.

If the drug is investigational (has an IND) or the device has an IDE or a claim of abbreviated IDE (non-significant risk device), include the following information:

- *Identify the holder of the IND/IDE/Abbreviated IDE.*
- *Explain procedures followed to comply with sponsor requirements for FDA regulated research for the following:*

	Applicable to:		
FDA Regulation	***IND Studies***	***IDE studies***	***Abbreviated IDE studies***
21 CFR 11	***X***	***X***	
21 CFR 54	***X***	***X***	
21 CFR 210	***X***		
21 CFR 211	***X***		
21 CFR 312	***X***		
21 CFR 812		***X***	***X***
21 CFR 820		***X***	

Response:

7.0 Local Number of Subjects

7.1 Indicate the total number of subjects that will be enrolled or records that will be reviewed locally.

Response:

50

7.2 If applicable, indicate how many subjects you expect to screen to reach your target sample (i.e. your screen failure rate).

Response:

50

7.3 Justify the feasibility of recruiting the proposed number of eligible subjects within the anticipated recruitment period. For example, how many potential subjects do you have access to? What percentage of those potential subjects do you need to recruit?

Response:

For the proposed dose-finding study, the 50 neuro long COVID patients enrolled in our earlier study will be contacted.

8.0 Inclusion and Exclusion Criteria*

8.1 Describe the criteria that define who will be ***included*** *in your final study sample.*

NOTE: This may be done in bullet point fashion.

Response:

Patient Eligibility Criteria for dose-finding extension study (Inclusion and Exclusion Criteria):

1) Enrolled from November 2022-July 2023 in lithium long COVID clinical trial at UB.
2) No fever or signs of acute infection for ≥4 weeks.
3) No COVID vaccine for ≥4 weeks.

4) Reports bothersome fatigue and/or brain fog while not taking lithium or, reports satisfactory benefit to these symptoms while taking lithium.
5) Fatigue Severity Scale (FSS) score ≥28 or Brain Fog Severity Scale (BFSS) score ≥28 at baseline or; FSS <28, BFSS <28 and PGIC at Visit 1 of "much improved" or "very much improved" while taking lithium.
6) Did not "respond" to placebo therapy, based on the responder analyses outlined in the Preliminary data section above, defined as a ≥18-point reduction FSS or ≥15-point reduction in BFSS from baseline to the end-of-double-blind study phase while receiving placebo therapy.
7) No change in any psychoactive or steroid medications for ≥30 days.
8) No plan to change any psychoactive, steroid or diuretic medication for ≥5 weeks.
9) No history of heart attack or stroke within the previous year.
10) No active medical, psychiatric or social problems that would interfere with completing the study procedures in the opinion of the investigator.
11) No daily NSAID use.
12) Not pregnant or nursing or planning to get pregnant over the next 11 weeks.

8.2 Describe the criteria that define who will be ***excluded*** *from your final study sample.*

NOTE: This may be done in bullet point fashion.

Response:

See 8.1.

8.3 Indicate specifically whether you will include any of the following special populations in your study using the checkboxes below.

NOTE: Members of special populations may not be targeted for enrollment in your study unless you indicate this in your inclusion criteria.

Response:

☐ Adults unable to consent

☐ Individuals who are not yet adults (infants, children, teenagers)

☐ Pregnant women

☐ Prisoners

8.4 Indicate whether you will include non-English speaking individuals in your study. ***Provide justification if you will exclude non-English speaking individuals.***

In order to meet one of the primary ethical principles of equitable selection of subjects, non-English speaking individuals may ***not*** *be routinely excluded from research as a matter of convenience.*

In cases where the research is of therapeutic intent or is designed to investigate areas that would necessarily require certain populations who

may not speak English, the researcher is required to make efforts to recruit and include non-English speaking individuals. However, there are studies in which it would be reasonable to limit subjects to those who speak English. Some examples include pilot studies, small unfunded studies with validated instruments not available in other languages, studies with numerous questionnaires, and some non-therapeutic studies which offer no direct benefit.

Response:

Only English-speaking individuals will be enrolled in this study as many of the study questionnaires are only available in English.

9.0 Vulnerable Populations*

If the research involves special populations that are considered vulnerable, ***describe the safeguards included to protect their rights and welfare.***

NOTE: You should refer to the appropriate checklists, referenced below, to ensure you have provided adequate detail regarding safeguards and protections. You do not, however, need to provide these checklists to the IRB.

9.1 For research that involves ***pregnant women,*** *safeguards include:*
NOTE CHECKLIST: Pregnant Women (HRP-412)

Response:

☒ **N/A**: This research does not involve pregnant women.

9.2 For research that involves ***neonates of uncertain viability or non-viable neonates,*** *safeguards include:*
NOTE CHECKLISTS: Non-Viable Neonates (HRP-413), or Neonates of Uncertain Viability (HRP-414)

Response:

☒ **N/A:** This research does not involve non-viable neonates or neonates of uncertain viability.

9.3 For research that involves ***prisoners****, safeguards include:*
NOTE CHECKLIST: Prisoners (HRP-415)

Response:

☒ **N/A:** This research does not involve prisoners.

9.4 For research that involves ***persons who have not attained the legal age for consent to treatments or procedures involved in the research ("children")****, safeguards include:*
NOTE CHECKLIST: Children (HRP-416)

Response:

☒ **N/A:** This research does not involve persons who have not attained the legal age for consent to treatments or procedures ("children").

*9.5 For research that involves **cognitively impaired adults**, safeguards include:*
NOTE CHECKLIST: Cognitively Impaired Adults (HRP-417)

Response:

☒ **N/A:** This research does not involve cognitively impaired adults.

*9.6 Consider if other specifically targeted populations such as students, employees of a specific firm, or educationally or economically disadvantaged persons are vulnerable. **Provide information regarding their safeguards and protections, including safeguards to eliminate coercion or undue influence.***

Response:

10.0 Eligibility Screening*

*10.1 Describe **screening procedures** for determining subjects' eligibility.*
Screening refers to determining if prospective participants meet inclusion and exclusion criteria.

Include all relevant screening documents with your submission (e.g. screening protocol, script, questionnaire).

Response:

Questionnaire data for patients enrolled in the original long COVID lithium RCT will be reviewed to screen for patient eligibility.

☐ **N/A:** There is no screening as part of this protocol.

11.0 Recruitment Methods

☐ **N/A:** This is a records review only, and subjects will not be recruited. NOTE: If you select this option, please make sure that all records review procedures and inclusion/exclusion screening are adequately described in other sections.

11.1 Describe when, where, and how potential subjects will be recruited.

NOTE: Recruitment refers to how you are identifying potential participants and introducing them to the study. Include specific methods you will use (e.g. searching charts for specific ICD code numbers, Research Participant Groups, posted advertisements, etc.).

Response:

For the dose-finding extension study:

A partial HIPAA waiver will be requested from the IRB in order to identify eligible patients from the original long COVID lithium RCT.

These patients will be contacted by email with the following text:
“Hello, I wanted to let you know of a new long COVID lithium research study being conducted at UBMD Neurology for which you may be eligible to participate. This new study seeks to determine if higher lithium dosages than previously studied are associated with more benefit in individual patients with long COVID. Attached is the study’s consent form for you to review. Let me know if you have any questions or interest in participating. Thank you.”

Patients who do not reply within 10 days will be mailed a letter with the same text as the email and a copy of the consent form. This will be the last time that patients are contacted if they do not reply.

11.2 *Describe how you will protect the privacy interests of prospective subjects during the recruitment process.*

NOTE: Privacy refers to an individual’s right to control access to him or herself.

Response:

For the dose-finding extension study:

Patients will only be contacted once by email and then once by mail. When discussing the study in the office, the PI will talk with potential participants in a private room.

11.3 *Identify any materials that will be used to recruit subjects.*

NOTE: Examples include scripts for telephone calls, in person announcements / presentations, email invitations.

For advertisements, include the final copy of printed advertisements with your submission. When advertisements are taped for broadcast, attach the final audio/video tape. NOTE: You may submit the wording of the advertisement prior to taping to ensure there will be no IRB-required revisions, provided the IRB also reviews and approves the final version.

Response:

For the dose-finding extension study:

A partial HIPAA waiver will be requested from the IRB in order to identify eligible patients from the original long COVID lithium RCT.

These patients will be contacted by email with the following text:
“Hello, I wanted to let you know of a new long COVID lithium research study being conducted at UBMD Neurology for which you may be eligible to participate. This new study seeks to determine if higher lithium dosages than previously studied are associated with more benefit in individual patients with

long COVID. Attached is the study's consent form for you to review. Let me know if you have any questions or interest in participating. Thank you."

Patients who do not reply within 10 days will be mailed a letter with the same text as the email and a copy of the consent form. This will be the last time that patients are contacted if they do not reply.

12.0 Procedures Involved*

12.1 Provide a description of ***all research procedures or activities*** *being performed and when they are performed once a subject is screened and determined to be eligible. Provide as much detail as possible.*

NOTE: This should serve as a blueprint for your study and include enough detail so that another investigator could pick up your protocol and replicate the research. For studies that have multiple or complex visits or procedures, consider the addition of a schedule of events table in in your response.

Response:

For the dose-finding extension study:

Baseline Visit (Visit 1):

- Written informed consent will be obtained after reviewing the study procedures and answering all of the patient's questions.
- Medical History focusing on long COVID symptoms and Personal Information (name, date of birth, gender, race/ethnicity, etc.) will be collected/confirmed.
- Review of symptoms and medications taken.
- Women of child-bearing potential will need to have a negative urine pregnancy test at this visit.
- Ten questionnaires will be completed: Headache and Body Pain Bother Scale, Generalized Anxiety Disorder Scale, Short Form-12 Health Survey, Fatigue Severity Scale, Modified Fatigue Impact Scale, Well Being Scale, Brain Fog Severity Scale, Perceived Deficits Questionnaire 5-Item Version, Beck Depression Inventory-II, and Insomnia Severity Index.
- Patients will receive a routine blood draw of about two teaspoonfuls of blood from an arm vein to assess lithium, thyroid and kidney function levels.
- Patients who are currently talking lithium and no longer bothered by long COVID symptoms will also receive a routine blood draw of about two teaspoonfuls of blood from an arm vein to assess trough lithium, thyroid and kidney function levels. The study will then be completed for these patients.
- This visit will take about 45 minutes.

Eligible patients will receive a supply of lithium aspartate 5mg capsules to be taken as follows: 2 capsules twice daily for one week, then 2 capsules every morning and 3 capsules at bedtime for one week, then 3 capsules twice daily for one week, then 3 capsules every morning and 4 capsules at bedtime for one week, then 4 capsules twice daily for one week, then 4 capsules every morning and 5 capsules at bedtime.

Patients who experience any side effects such as sleepiness or dizziness will be instructed to call Dr. Guttuso at 716-324-3247 to inform him. The lithium dosage will be

decreased by one capsule/day to help resolve these side effects. Dr. Guttuso will call the patient 2-3 days later to determine if the dosage needs to be further reduced. Patients who experience satisfactory improvements to their long COVID symptoms as the lithium dosage is increased will be instructed to stay at that dosage and not increase it further. Patients will be informed that the goal is to find the lowest lithium dosage that provides satisfactory benefits for them. If a patient experiences satisfactory benefit at a particular lithium dosage for at least two weeks, they will be instructed to call Dr. Guttuso at 716-324-3247 or 716-932-6080, ext. 135 to schedule Visit 2. Patients who do not experience satisfactory benefit at nine lithium capsules/day or who experience side effects preventing dose escalation will also be scheduled for Visit 2.

Study Visit (Visit 2)– performed about 6-11 weeks after Visit 1

- Side effects will be assessed and current lithium dosage confirmed.
- Concomitant medications and medical problems will be reviewed.
- The same questionnaires from Visit 1 will be completed in addition to the Patient Global Impression of Change (PGIC) scale and the Desire to Continue Therapy Scale.
- A routine blood draw will be performed of about two teaspoonfuls of blood from an arm vein to assess trough lithium, thyroid and kidney function levels.
- This visit will take about 45 minutes.

Patients who complete the above two study visits will be offered lithium aspartate capsules to take for three additional weeks at the same dosage they were most recently taking. After three weeks of lithium treatment, patients will be asked to complete provided questionnaires from Visit 2 and mail them to the study team in a self-addressed stamped envelope. This will end the study. Interested patients will be provided with information on how to continue lithium aspartate therapy after the study.

12.2 Describe what data will be collected.

NOTE: For studies with multiple data collection points or long-term follow up, consider the addition of a schedule or table in your response.

Response:

Patient medical history, demographic information, questionnaires and blood tests.

12.3 List any instruments or measurement tools used to collect data (e.g. questionnaire, interview guide, validated instrument, data collection form).

Include copies of these documents with your submission.

Response:

The Headache and Body Pain Bother Scale, the Generalized Anxiety Disorder Scale (GAD-2), the Short Form-12 Health Survey (SF-12 quality of life survey), the Fatigue Severity Scale, the Well Being Scale, the Brain Fog Severity Scale, the Beck Depression Inventory-II (BDI-II), the Insomnia Severity Index, the Patient Global Impression of Change (PGIC) scale, Modified Fatigue Impact Scale, Perceived Deficits Questionnaire 5-Item Version and the Desire to Continue Therapy Scale.

12.4 Describe any source records that will be used to collect data about subjects (e.g. school records, electronic medical records).

Response:

No source records will be used to collect data for the dose-finding extension study except for the records collected during the original long COVID lithium study.

*12.5 Indicate whether or not **individual** subject results, such as results of investigational diagnostic tests, genetic tests, or incidental findings will be shared with subjects or others (e.g., the subject's primary care physician) and if so, describe how these will be shared.*

Response:

Individual patients will be informed of their treatment allocation by mail after the study is completed.

*12.6 Indicate whether or not **study** results will be shared with subjects or others, and if so, describe how these will be shared.*

Response: Individual patients will be not be informed of the study results. The final results from this study will be presented in medical meetings and published in a medical journal.

13.0 Study Timelines*

13.1 Describe the anticipated duration needed to enroll all study subjects.

Response:

20 weeks for the dose-finding extension study.

13.2 Describe the duration of an individual subject's participation in the study. Include length of study visits, and overall study follow-up time.

Response:

For the dose-finding extension study, each of the two study visits will take about 45 minutes. Each patient will be in the study for 6-11 weeks depending on which lithium dosage provides satisfactory benefit to the patient's neuro long COVID symptoms, per the above dose titration schedule.

13.3 Describe the estimated duration for the investigators to complete this study (i.e. all data is collected and all analyses have been completed).

Response:

40 weeks

14.0 Setting

14.1 Describe all facilities/sites where you will be conducting research procedures. Include a description of the security and privacy of the

facilities (e.g. locked facility, limited access, privacy barriers). Facility, department, and type of room are relevant. Do not abbreviate facility names.

NOTE: Examples of acceptable response may be: "A classroom setting in the Department of Psychology equipped with a computer with relevant survey administration software," "The angiogram suite at Buffalo General Medical Center, a fully accredited tertiary care institution within New York State with badge access," or, "Community Center meeting hall."

Response:

All clinical visits will occur at UBMD Neurology at 5851 Main Street, Williamsville, NY 14221. This is a clinic where neurology patients are routinely treated. The facility is staffed during business hours and otherwise locked.

14.2 For research conducted outside of UB and its affiliates, describe:

- *Site-specific regulations or customs affecting the research*
- *Local scientific and ethical review structure*

NOTE: This question is referring to UB affiliated research taking place outside UB, i.e. research conducted in the community, school-based research, international research, etc. It is not referring to multi-site research. UB affiliated institutions include Kaleida Health, ECMC, and Roswell Park Cancer Institute.

Response:

☒ **N/A:** This study is not conducted outside of UB or its affiliates.

15.0 Community-Based Participatory Research

15.1 Describe involvement of the community in the design and conduct of the research.

NOTE: Community-Based Participatory Research (CBPR) is a collaborative approach to research that equitably involves all partners in the research process and recognizes the unique strengths that each brings. CBPR begins with a research topic of importance to the community, has the aim of combining knowledge with action and achieving social change to improve health outcomes and eliminate health disparities.

Response:

☒ **N/A:** This study does not utilize CBPR.

15.2 Describe the composition and involvement of a community advisory board.

Response:

☒ **N/A:** This study does not have a community advisory board.

16.0 Resources and Qualifications

*16.1 Describe the qualifications (e.g., education, training, experience, expertise, or certifications) of the Principal Investigator **and** staff to perform the research. When applicable describe their knowledge of the local study sites, culture, and society. Provide enough information to convince the IRB that you have qualified staff for the proposed research.*

NOTE: If you specify a person by name, a change to that person will require prior approval by the IRB. If you specify a person by role (e.g., coordinator, research assistant, co-investigator, or pharmacist), a change to that person will not usually require prior approval by the IRB, provided that the person meets the qualifications described to fulfill their roles.

Response:

The PI, Thomas Guttuso, Jr., MD is a Professor of Neurology at UB. Dr. Guttuso has served as PI on nine, hypothesis-driven clinical trials and site-PI on three, industry-sponsored clinical trials. The study coordinator has over 3 years of experience in this role on clinical trials.

Describe other resources available to conduct the research.

16.2 Describe the time and effort that the Principal Investigator and research staff will devote to conducting and completing the research.

NOTE: Examples include the percentage of Full Time Equivalents (FTE), hours per week. The question will elicit whether there are appropriate resources to conduct the research.

Response:

The PI will devote 0.2FTE and the coordinator 0.2FTE towards this project.

16.3 Describe the availability of medical or psychological resources that subjects might need as a result of anticipated consequences of the human research, if applicable.

NOTE: One example includes: on-call availability of a counselor or psychologist for a study that screens subjects for depression.

Response: N/A

16.4 Describe your process to ensure that all persons assisting with the research are adequately informed about the protocol, the research procedures, and their duties and functions.

Response:

The PI will directly communicate with all study staff to review study procedures and define each member’s duties. The PI will monitor all staff throughout the study to ensure that study procedures are being followed appropriately.

17.0 Other Approvals

17.1 Describe any approvals that will be obtained prior to commencing the research (e.g., school, external site, funding agency, laboratory, radiation safety, or biosafety).

Response:

☒ **N/A:** This study does not require any other approvals.

18.0 Provisions to Protect the Privacy Interests of Subjects

18.1 Describe how you will protect subjects' privacy interests during the course of this research.

NOTE: Privacy refers to an individual's right to control access to him or herself. Privacy applies to the person. Confidentiality refers to how data collected about individuals for the research will be protected by the researcher from release. Confidentiality applies to the data.

Examples of appropriate responses include: "participant only meets with a study coordinator in a classroom setting where no one can overhear", or "the participant is reminded that they are free to refuse to answer any questions that they do not feel comfortable answering."

Response:

Study subjects will meet with either the PI or coordinator in a clinic room with the door shut for privacy.

18.2 Indicate how the research team is permitted to access any sources of information about the subjects.

NOTE: Examples of appropriate responses include: school permission for review of records, consent of the subject, HIPAA waiver. This question ***does apply*** *to records reviews.*

Response:

Consent of the subject.

19.0 Data Management and Analysis*

19.1 Describe the data analysis plan, including any statistical procedures. This section applies to both quantitative and qualitative analysis.

Response:

For the dose-finding extension study, the "two groups" that will be compared are the change from baseline to end of open-label lithium 15mg/day treatment from the original trial and the change from baseline to end of open-label lithium 20-45mg/day treatment from this open-label dose-finding extension trial for each individual patient. To describe the observed variability in the data and test for differences between the groups, a linear model will be fit to each considered outcome. Specifically, each numeric outcome will be modeled as a function of each group and the baseline value of the outcome variable. Once the model is fit, a linear contrast based on the estimated model parameters will be constructed and used to test for the overall effect of random assignment using an approximate F-test as implemented by SAS PROC MIXED. A point estimate and corresponding confidence interval to quantify treatment differences will be provided. All tests will be two-sided and tested at a 0.05 nominal significance level. Standard diagnostic plots will be used to assess model fit and transformations of variables may be considered in order to meet statistical assumptions.

19.2 *If applicable, provide a power analysis.*

NOTE: This may not apply to certain types of studies, including chart/records reviews, survey studies, or observational studies. This question is asked to elicit whether the investigator has an adequate sample size to achieve the study objectives and justify a conclusion.

Response:

Power analysis is not indicated for the dose-finding extension study.

19.3 *Describe any procedures that will be used for quality control of collected data.*

Response:

After patients complete the questionnaires, the PI will review each to confirm that all questions were answered.

20.0 Confidentiality*

A. Confidentiality of Study Data

Describe the local procedures for maintenance of confidentiality of ***study data and any records that will be reviewed for data collection****.*

20.1 A. Where and how will all data and records be stored? Include information about: password protection, encryption, physical controls, authorization of access, and separation of identifiers and data, as applicable. Include physical (e.g. paper) ***and*** *electronic files.*

Response:

A study binder will be assembled for each subject that will contain all of their study data. However, the forms linking subjects' personal identifiable information to their study ID numbers will be kept in a separate folder. This folder and all study binders will be stored in a locked room in a locked cabinet at the study site. These data will be entered into an excel file in a password protected computer located in this same locked room. The excel file will only contain subject ID numbers and no personal identifiable information.

20.2 A. How long will the data be stored?

Response:

IRB correspondences and signed consent forms will be kept for at least three years. Patient identifiers will be deleted at the first available opportunity after it is no longer necessary to conduct the research. Data analysis files that contain no dates or patient identifiers will be kept indefinitely.

20.3 A. Who will have access to the data?

Response:

The PI, study coordinator and statistician.

20.4 A. Who is responsible for receipt or transmission of the data?

Response:

The PI.

20.5 A. How will the data be transported?

Response:

Electronically.

B. Confidentiality of Study Specimens

*Describe the local procedures for maintenance of confidentiality of **study specimens**.*

☐ **N/A:** No specimens will be collected or analyzed in this research. *(Skip to Section 21.0)*

20.6 B. Where and how will all specimens be stored? Include information about: physical controls, authorization of access, and labeling of specimens, as applicable.

Response:

Blood samples will be transported to Kaleida laboratory for processing the day they are collected.

20.7 B. How long will the specimens be stored?

Response:

1 day.

20.8 B. Who will have access to the specimens?

Response:

The PI, coordinator and Kaleida laboratory technician.

20.9 B. Who is responsible for receipt or transmission of the specimens?

Response:

The PI.

20.10 B. How will the specimens be transported?

Response:

By a Kaleida courier.

21.0 Provisions to Monitor the Data to Ensure the Safety of Subjects*

☐ **N/A:** This study is not enrolling subjects, or is limited to records review procedures only. This section does not apply.

NOTE: Minimal risk studies may be required to monitor subject safety if the research procedures include procedures that present unique risks to subjects that require monitoring. Some examples include: exercising to exertion, or instruments that elicit suicidality or substance abuse behavior. In such cases, N/A is not an acceptable response.

21.1 Describe the plan to periodically evaluate the data collected regarding both harms and benefits to determine whether subjects remain safe.

Response:

Enrolled patients will be provided with the study teams' contact information as well as the UBMD Neurology call service phone number to be used on weekends and after business hours during the week in order to report any adverse events they may be experiencing during the study. If patients experience any adverse events, the lithium dosage will be decreased until side effects resolve.

21.2 Describe what data are reviewed, including safety data, untoward events, and efficacy data.

Response:

Patient adverse events.

21.3 Describe any safety endpoints.

Response:

Total number of adverse events and individual adverse events.

21.4 Describe how the safety information will be collected (e.g., with case report forms, at study visits, by telephone calls with participants).

Response:

Adverse events will be collected by phone and inquired about at Visit 2.

21.5 Describe the frequency of safety data collection.

Response:

The only formal inquiry of adverse events will be at Visit 2. However, patients can contact the study team by phone at any time during the study to report adverse events.

21.6 Describe who will review the safety data.

Response:

The PI will review AEs monthly. The safety monitor, Daniel Sirica, MD, will review AEs quarterly.

21.7 Describe the frequency or periodicity of review of cumulative safety data.

Response:

Monthly.

21.8 Describe the statistical tests for analyzing the safety data to determine whether harm is occurring.

Response:

None.

21.9 Describe any conditions that trigger an immediate suspension of the research.

Response:

- For <u>non-serious</u> subject adverse events:
  Subjects will be directly asked by one of the study team members about any adverse events at Visit 2. All adverse events (AE's) will be recorded on the AE report form. Non-serious adverse events will be categorized as follows:
  - **Mild** – Events require minimal or no treatment and do not interfere with the participant's daily activities.
  - **Moderate** – Events result in a low level of inconvenience or concern with the therapeutic measures. Moderate events may cause some interference with functioning.
  - **Severe** – Events interrupt a participant's usual daily activity and may require systemic drug therapy or other treatment. Severe events are usually potentially life-threatening or incapacitating. Of note, the term "severe" does not necessarily equate to "serious".

- For <u>serious</u> subject adverse events (SAE):
  An adverse event will be considered "serious" (SAE) if the adverse event resulted in subject death, a life-threatening adverse event, inpatient hospitalization or prolongation of existing hospitalization, or a persistent or significant incapacity or substantial disruption of the ability to conduct normal life functions. This is the definition of SAE according to the FDA's Guidance for Industry and Investigators: Safety Reporting Requirements for INDs and BA/BE Studies. When a study team member becomes aware of a possible subject SAE, the study team member will contact the PI by phone the same day.
  An SAE that is determined by the study PI to be, a) unexpected, and b) reasonably possible to have been caused by lithium will be considered a "Reportable Event". The PI will report such events to the University at Buffalo's IRB within 7 calendar days of the study team's first awareness of the event.
  An SAE that is unexpected by virtue of it not being listed in either the FDA approved product package insert, protocol or consent form with regard to occurrence or at an increased frequency, severity or duration than previously published will be considered a "Reportable Event". FDA Guidance from 2012 notes that an event should be reported if:

-A single occurrence of an event that is uncommon and known to be strongly associated with drug exposure (e.g., angioedema, hepatic injury, Stevens-Johnson Syndrome)

-One or more occurrences of an event that is not commonly associated with drug exposure, but is otherwise uncommon in the population exposed to the drug (e.g., tendon rupture)

-An aggregate analysis of specific events observed in a clinical trial (such as known consequences of the underlying disease or condition under investigation or other events that commonly occur in the study population independent of drug therapy) that indicates those events occur more frequently in the drug treatment group than in a concurrent or historical control group.

- If similar "Reportable" SAEs occur in five subjects, the statistician will be unblinded to the treatment allocation for these subjects and alert the PI if all five are receiving lithium. If so, the study will be terminated and subjects instructed to stop lithium therapy.
- No interim data analysis will be performed.
- This study will be registered on clinicaltrials.gov prior to subject enrollment.

22.0 Withdrawal of Subjects*

☐ **N/A:** This study is not enrolling subjects. This section does not apply.

22.1 Describe ***anticipated*** *circumstances under which subjects may be withdrawn from the research without their consent.*

Response:

Subjects experiencing a "reportable" SAE (defined above) will be withdrawn.

22.2 Describe any procedures for orderly termination.

NOTE: Examples may include return of study drug, exit interview with clinician. Include whether additional follow up is recommended for safety reasons for physical or emotional health.

Response:

Subjects withdrawn from the study will be asked to make a final study visit during which all Visit 2 procedures will take place. Subjects withdrawn after visit 2 will be asked to complete the questionnaires at home and mail them to the study team in the provided self-addressed, stamped envelope.

22.3 Describe procedures that will be followed when subjects withdraw from the research, including retention of already collected data, and partial withdrawal from procedures with continued data collection, as applicable.

Response:

All data provided by subjects withdrawn from the study will be retained and used in the final analysis.

23.0 Risks to Subjects*

23.1 List the reasonably foreseeable risks, discomforts, hazards, or inconveniences to the subjects related to their participation in the research. Consider physical, psychological, social, legal, and economic risks. Include a description of the probability, magnitude, duration, and reversibility of the risks.

NOTE: Breach of confidentiality is always a risk for identifiable subject data.

Response:

Patients may experience sleepiness or dizziness as they increase the lithium dosage. It is unlikely that the low lithium dosages being used in this study will cause thyroid or kidney dysfunction. Thyroid and kidney function blood tests will be monitored at both study visits.

Questionnaires: filling out the questionnaires or answering the study doctor or study staff's questions could lead patients to feel uncomfortable or upset.

Venipucture can cause discomfort and lightheadedness in a patient and swelling, bruising or infection at the puncture site.

23.2 Describe procedures performed to lessen the probability or magnitude of risks, including procedures being performed to monitor subjects for safety.

Response:

If a patient experiences side effects at any lithium dosage, the dose can be decreased until the side effects resolve.

Patients will be instructed to tell the study doctor or study staff if they feel uncomfortable or upset while filling out a questionnaire or answering questions so that they can be counselled and their concerns addressed.

Patients will be asked if they prefer to lay down for the venipuncture. Sterile techniques will be used by the nurse performing the venipuncture.

23.3 If applicable, indicate ***which procedures*** *may have risks to the subjects that are currently unforeseeable.*

Response:

Since the study drug is investigational for treating long COVID, there may be other risks that are unknown. Patients will be informed in a timely manner if new information about the study becomes available that may be relevant to continued participation in the trial. All drugs have a potential risk of an allergic reaction, which if not treated promptly, could become life-threatening. Some things that happen during an allergic reaction that could be a sign or symptom of a life-threatening allergic reaction (anaphylaxis) are:

- a rash
- a fast pulse

- sweating
- a feeling of dread
- swelling around the eyes and mouth
- swelling of the throat
- wheezing
- having a hard time breathing
- a sudden drop in blood pressure (making you feel dizzy or lightheaded)
- inability to breathe without assistance

Patients will be informed to get medical help and contact the study doctor or study staff if they have any of these or any other side effects during the study.

23.4 If applicable, indicate which research procedures may have risks to an embryo or fetus should the subject be or become pregnant.

Response:

Lithium therapy is associated with higher rates of birth defects. Therefore, women of child bearing potential must have a negative pregnancy test to be eligible for this study. In order to reduce the risk of pregnancy during the study, women of child bearing potential must use an effective method of birth control while participating in this study. Acceptable methods of birth control include oral contraceptive or long-term injectable or implantable hormonal contraceptive, double-barrier methods such as condom plus diaphragm, condom plus spermicide foam, condom plus sponge, or intra-uterine devices or abstinence. Oral, implantable, or injectable contraceptives are only considered effective if used properly and started at least 30 days prior to the screening visit. Some drugs (e.g., antibiotics) may interact with hormonal contraceptives, making them not work properly.

23.5 If applicable, describe risks to others who are not subjects.

Response:

None.

24.0 Potential Benefits to Subjects*

24.1 Describe the potential benefits that individual subjects may experience by taking part in the research. Include the probability, magnitude, and duration of the potential benefits. Indicate if there is no direct benefit.

*NOTE: Compensation **cannot** be stated as a benefit.*

Response:

Patients may notice improvements in their long COVID symptoms.

25.0 Compensation for Research-Related Injury

☐ **N/A:** The research procedures for this study do not present risk of research related injury (e.g. survey studies, records review studies). This section does not apply.

25.1 ***If the research procedures carry a risk of research related injury,*** *describe the available compensation to subjects in the event that such injury should occur.*

Response:

Subjects will receive medical treatment if injured or become ill as a result of this study.

The University at Buffalo makes no commitment to provide free medical care or payment for any unfavorable outcomes that result from your participation in this research. Medical services will be billed at the usual charge and will be the subject's responsibility or that of a third-party payer but subjects will not be precluded from seeking to collect compensation for injury related to malpractice, fault, or blame on the part of those involved in the research including the University at Buffalo.

By accepting medical care or accepting payment for medical expenses, subjects are not waiving any legal rights.

25.2 *Provide a copy of contract language, if any, relevant to compensation for research related injury.*

NOTE: If the contract is not yet approved at the time of this submission, submit the current version here. If the contract is later approved with ***different language regarding research related injury****, you must modify your response here and submit an amendment to the IRB for review and approval.*

Response:

N/A

26.0 Economic Burden to Subjects

26.1 *Describe any costs that subjects may be responsible for because of participation in the research.*

NOTE: Some examples include transportation or parking.

Response:

Transportation costs. Parking is free.

☐ **N/A:** This study is not enrolling subjects, or is limited to records review procedures only. This section does not apply.

27.0 Compensation for Participation

27.1 *Describe the amount and timing of any compensation to subjects, including monetary, course credit, or gift card compensation.*

Response:

☐ **N/A:** This study is not enrolling subjects, or is limited to records review procedures only. This section does not apply.

☒ **N/A:** There is no compensation for participation. This section does not apply.

28.0 Consent Process

28.1 Indicate whether you will be obtaining consent.

NOTE: This does not refer to consent documentation, but rather whether you will be obtaining permission from subjects to participate in a research study. Consent documentation is addressed in Section 29.0.

☒ ***Yes*** *(If yes, Provide responses to each question in this Section)*
☐ ***No*** *(If no, Skip to Section 29.0)*

28.2 Describe where the consent process will take place. Include steps to maximize subjects' privacy.

Response:

The consent process will occur in a closed-door, examination clinic room at UBMD Neurology.

28.3 Describe how you will ensure that subjects are provided with a sufficient period of time to consider taking part in the research study.

NOTE: It is always a requirement that a prospective subject is given sufficient time to have their questions answered and consider their participation. See "SOP: Informed Consent Process for Research (HRP-090)" Sections 5.5 and 5.6.

Response:

Potential subjects will be provided with the consent document at least 48 hours prior to the screening visit to ensure adequate time to read and process the entire document.

28.4 Describe any process to ensure ongoing consent, defined as a subject's willingness to continue participation for the duration of the research study.

Response:

Subjects will be asked if they wish to continue in the study at each study visit and phone call.

28.5 Indicate whether you will be following "SOP: Informed Consent Process for Research (HRP-090)." Pay particular attention to Sections 5.4-5.9. If not, or if there are any exceptions or additional details to what is covered in the SOP, describe:

- *The role of the individuals listed in the application who are involved in the consent process*
- *The time that will be devoted to the consent discussion*
- *Steps that will be taken to minimize the possibility of coercion or undue influence*
- *Steps that will be taken to ensure the subjects' understanding*

Response:

☒ We have reviewed and will be following "SOP: Informed Consent Process for Research (HRP-090)."

Non-English Speaking Subjects

☒ **N/A:** This study will not enroll Non-English speaking subjects. *(Skip to Section 28.8)*

28.6 *Indicate which language(s) other than English are likely to be spoken/understood by your prospective study population or their legally authorized representatives.*

NOTE: The response to this Section should correspond with your response to Section 8.4 of this protocol.

Response:

28.7 *If subjects who do not speak English will be enrolled, describe the process to ensure that the oral and written information provided to those subjects will be in that language, how you will ensure that subjects are provided with a sufficient period of time to consider taking part in the research study, and any process to ensure ongoing consent. Indicate the language that will be used by those obtaining consent.*

NOTE: Guidance is provided on "SOP: Informed Consent Process for Research (HRP-090)."

Response:

Cognitively Impaired Adults

☐ **N/A**: This study will not enroll cognitively impaired adults. *(Skip to Section 28.9)*

28.8 *Describe the process to determine whether an individual is capable of consent.*

Response:

After subjects have had adequate time to review the consent and ask questions about the study, they will be asked to give a brief overview of the study procedures. Only subjects that can accurately describe key aspects of the study will be enrolled.

Adults Unable to Consent

☒ **N/A**: This study will not enroll adults unable to consent.
(*Skip to Section 28.13)*

When a person is not capable of consent due to cognitive impairment, a legally authorized representative should be used to provide consent (Sections 28.9 and 28.10) ***and, where possible, assent of the individual should also be solicited*** *(Sections 28.11 and 28.12).*

28.9 Describe how you will identify a Legally Authorized Representative (LAR). Indicate that you have reviewed the "SOP: Legally Authorized Representatives, Children, and Guardians (HRP-013)" for research in New York State.

NOTE: Examples of acceptable response includes: verifying the electronic medical record to determine if an LAR is recorded.

Response:

☐ We have reviewed and will be following "SOP: Legally Authorized Representatives, Children, and Guardians (HRP-013)."

28.10 ***For research conducted outside of New York State****, provide information that describes which individuals are authorized under applicable law to consent on behalf of a prospective subject to their participation in the research. One method of obtaining this information is to have a legal counsel or authority review your protocol along with the definition of "legally authorized representative" in "SOP: Legally Authorized Representatives, Children, and Guardians (HRP-013)."*

Response:

28.11 Describe the process for ***assent of the adults****:*

- *Indicate whether assent will be obtained from all, some, or none of the subjects.* ***If some, indicate which adults will be required to assent and which will not.***

Response:

- ***If assent will not be obtained from some or all subjects, provide an explanation of why not.***

Response:

28.12 Describe whether ***assent of the adult*** *subjects will be documented and the process to document assent.*

NOTE: The IRB allows the person obtaining assent to document assent on the consent document using the "Template Consent Document (HRP-502)" Signature Block for Assent of Adults who are Legally Unable to Consent.

Response:

Subjects who are not yet Adults (Infants, Children, and Teenagers)

☒ **N/A**: This study will not enroll subjects who are not yet adults. *(Skip to Section 29.0)*

*28.13 Describe the criteria that will be used to determine **whether a prospective subject has not attained the legal age for consent to treatments or procedures involved in the research** under the applicable law of the jurisdiction in which the research will be conducted **(e.g., individuals under the age of 18 years)**. For research conducted in NYS, review "SOP: Legally Authorized Representatives, Children, and Guardians (HRP-013)" to be aware of which individuals in the state meet the definition of "children."*

NOTE: Examples of acceptable responses include: verification via electronic medical record, driver's license or state-issued ID, screening questionnaire.

Response:

*28.14 **For research conducted outside of New York State**, provide information that describes which persons have not attained the legal age for consent to treatments or procedures involved the research, under the applicable law of the jurisdiction in which research will be conducted. One method of obtaining this information is to have a legal counsel or authority review your protocol along the definition of "children" in "SOP: Legally Authorized Representatives, Children, and Guardians (HRP-013)."*

28.15 Describe whether parental permission will be obtained from:

Response:

☐ One parent even if the other parent is alive, known, competent, reasonably available, and shares legal responsibility for the care and custody of the child.

☐ Both parents unless one parent is deceased, unknown, incompetent, or not reasonably available, or when only one parent has legal responsibility for the care and custody of the child.

☐ Parent permission will not be obtained. A waiver of parent permission is being requested.

NOTE: The requirement for parent permission is a protocol-specific determination made by the IRB based on the risk level of the research. For guidance, review the "CHECKLIST: Children (HRP-416)."

28.16 Describe whether permission will be obtained from individuals ***other than parents****, and if so, who will be allowed to provide permission. Describe your procedure for determining an individual's authority to consent to the child's general medical care.*

Response:

28.17 Indicate whether assent will be obtained from all, some, or none of the ***children****. If assent will be obtained from some children, indicate which children will be required to assent.*

Response:

28.18 When assent of children is obtained, describe how it will be documented.

Response:

29.0 Waiver or Alteration of Consent Process

Consent will not be obtained, required information will not be disclosed, or the research involves deception.

☒ **N/A:** A waiver or alteration of consent is not being requested.

29.1 If the research involves a waiver or alteration of the consent process, please review the "CHECKLIST: Waiver or Alteration of Consent Process (HRP-410)" to ensure that you have provided sufficient information for the IRB to make the determination that a waiver or alteration can be granted.

NOTE: For records review studies, the first set of criteria on the "CHECKLIST: Waiver or Alteration of Consent Process (HRP-410)" applies.

Response:

29.2 If the research involves a waiver of the consent process for planned emergency research, please review the "CHECKLIST: Waiver of Consent for Emergency Research (HRP-419)" to ensure you have provided sufficient information for the IRB to make these determinations. Provide any additional information necessary here:

Response:

30.0 Process to Document Consent

☐ **N/A:** A Waiver of Consent is being requested.
(Skip to Section 31.0)

30.1 Indicate whether you will be following "SOP: Written Documentation of Consent (HRP-091)." If not or if there are any exceptions, describe whether and how consent of the subject will be obtained including whether or not it will be documented in writing.

NOTE: If your research presents no more than minimal risk of harm to subjects and involves no procedures for which written documentation of consent is normally required outside of the research context, the IRB will generally waive the requirement to obtain written documentation of consent. This is sometimes referred to as 'verbal consent.' Review "CHECKLIST: Waiver of Written Documentation of Consent (HRP-411)" to ensure that you have provided sufficient information.

If you will document consent in writing, attach a consent document with your submission. You may use "TEMPLATE CONSENT DOCUMENT (HRP-502)". If you will obtain consent, but not document consent in writing, attach the script of the information to be provided orally or in writing (i.e. consent script or Information Sheet).

Response:

☒ We will be following "SOP: Written Documentation of Consent" (HRP-091).

31.0 Multi-Site Research (Multisite/Multicenter Only)*

☒ **N/A:** This study is not an investigator-initiated multi-site study. This section does not apply.

31.1 Indicate the total number of subjects that will be enrolled or records that will be reviewed across all sites.

Response:

*31.2 If this is a multi-site study **where you are the lead investigator**, describe the processes to ensure communication among sites, such as the following.*

- *All sites have the most current version of the IRB documents, including the protocol, consent document, and HIPAA authorization.*
- *All required approvals have been obtained at each site (including approval by the site's IRB of record).*
- *All modifications have been communicated to sites, and approved (including approval by the site's IRB of record) before the modification is implemented.*

- *All engaged participating sites will safeguard data as required by local information security policies.*
- *All local site investigators conduct the study appropriately in accordance with applicable federal regulations and local laws.*
- *All non-compliance with the study protocol or applicable requirements will be reported in accordance with local policy.*

Response:

31.3 Describe the method for communicating to engaged participating sites.

- *Problems (inclusive of reportable events)*
- *Interim results*
- *Study closure*

Response:

31.4 If this is a multicenter study ***where you are a participating site/investigator****, describe the local procedures for maintenance of confidentiality.*

- *Where and how data or specimens will be stored locally?*
- *How long the data or specimens will be stored locally?*
- *Who will have access to the data or specimens locally?*
- *Who is responsible for receipt or transmission of the data or specimens locally?*
- *How data and specimens will be transported locally?*

Response:

31.5 If this is a multicenter study and subjects will be recruited by methods not under the control of the local site (e.g., call centers, national advertisements) describe those methods. Local recruitment methods are described elsewhere in the protocol.

- *Describe when, where, and how potential subjects will be recruited.*
- *Describe the methods that will be used to identify potential subjects.*
- *Describe materials that will be used to recruit subjects. (Attach copies of these documents with the application. For advertisements, attach the final copy of printed advertisements. When advertisements are taped for broadcast, attach the final audio/video tape. You may submit the wording of the advertisement prior to taping to preclude re-taping because of*

inappropriate wording, provided the IRB reviews the final audio/video tape.)

Response:

32.0 Banking Data or Specimens for Future Use*

☐ **N/A:**

32.1 *If data or specimens will be banked (stored) for* ***future use, that is, use or research outside of the scope of the present protocol****, describe where the data/specimens will be stored, how long they will be stored, how the data/specimens will be accessed, and who will have access to the data/specimens.*

NOTE: Your response here must be consistent with your response at the "What happens if I say yes, I want to be in this research?" Section of the Template Consent Document (HRP-502).

NOTE: If the UBIRB has approved this study to bank data and/or specimens for potential future use outside the scope of this research study, any future use or disclosure of the data that is not described within the approved study must be submitted for review to the UBIRB.

Response: Patient deidentified data will be stored in a password-protected excel file indefinitely. The study PI will have access to the data.

32.2 *List the data to be stored or associated with each specimen.*

Response: Deidentified questionnaire, blood test and patient demographic data.

32.3 *Describe the procedures to release banked data or specimens for future uses, including: the process to request a release, approvals required for release, who can obtain data or specimens, and the data to be provided with specimens.*

Response: The PI will grant data access upon reasonable written request from a reputable research team.
