# Supplementary material for: Lithium Aspartate for Long COVID Fatigue and Cognitive Dysfunction: A Randomized Clinical Trial
Source: JAMA Netw Open. 2024 Oct 2;7(10):e2436874. doi: 10.1001/jamanetworkopen.2024.36874 (PMC11447566; doi:10.1001/jamanetworkopen.2024.36874)
Supplement: Supplement 3. — Data Sharing Statement [file jamanetwopen-e2436874-s003.pdf]

Data Sharing Statement

Guttuso, Jr. Lithium Aspartate for Long COVID Fatigue and Cognitive Dysfunction. *JAMA Netw Open*. Published October 02, 2024. doi:10.1001/jamanetworkopen.2024.36874

Data
Data available: Yes
Data types: Deidentified participant data
How to access data: tguttuso@buffalo.edu
When available: With publication

Supporting Documents
Document types: None

Additional Information
Who can access the data: Researchers whose proposed use of the data has been approved.
Types of analyses: Any approved secondary analyses.
Mechanisms of data availability: After approval of a proposal.
Any additional restrictions: None.
